# Myrtucommulones and Related Acylphloroglucinols from Myrtaceae as a Promising Source of Multitarget SARS-CoV-2 Cycle Inhibitors

**DOI:** 10.3390/ph17040436

**Published:** 2024-03-28

**Authors:** Simony Carvalho Mendonça, Brendo Araujo Gomes, Mariana Freire Campos, Thamirys Silva da Fonseca, Maria Eduarda Alves Esteves, Bruce Veiga Andriolo, Caio Felipe de Araujo Ribas Cheohen, Larissa Esteves Carvalho Constant, Stephany da Silva Costa, Pedro Telles Calil, Amanda Resende Tucci, Thamara Kelcya Fonseca de Oliveira, Alice dos Santos Rosa, Vivian Neuza dos Santos Ferreira, Julia Nilo Henrique Lima, Milene Dias Miranda, Luciana Jesus da Costa, Manuela Leal da Silva, Marcus Tullius Scotti, Diego Allonso, Gilda Guimarães Leitão, Suzana Guimarães Leitão

**Affiliations:** 1 Departamento de Produtos Naturais e Alimentos, Faculdade de Farmácia, Universidade Federal do Rio de Janeiro, Rio de Janeiro 21941-902, RJ, Brazil; sy2802@ufrj.br (S.C.M.); brendoo.bc@ufrj.br (B.A.G.); camposmariana@biof.ufrj.br (M.F.C.); 2 Programa de Pós-Graduação em Biotecnologia Vegetal e Bioprocessos, Centro de Ciências da Saúde, Universidade Federal do Rio de Janeiro, Rio de Janeiro 21941-902, RJ, Brazil; 3 Programa de Pós-Graduação em Ciências Farmacêuticas, Faculdade de Farmácia, Universidade Federal do Rio de Janeiro, Rio de Janeiro 21941-902, RJ, Brazil; thamirysfonseca@ufrj.br; 4 Programa de Pós-Graduação em Biologia Computacional e Sistemas, Instituto Oswaldo Cruz, Rio de Janeiro 21040-900, RJ, Brazil; mariaesteves@aluno.fiocruz.br (M.E.A.E.); manuela@macae.ufrj.br (M.L.d.S.); 5 Programa de Pós-Graduação em Biotecnologia, Instituto Nacional de Metrologia, Qualidade e Tecnologia, Duque de Caxias 25250-020, RJ, Brazil; bruceandriolo@ufrj.br; 6 Programa de Pós-Graduação Multicêntrico em Ciências Fisiológicas, Centro de Ciências da Saúde, Instituto de Biodiversidade e Sustentabilidade NUPEM, Universidade Federal do Rio de Janeiro, Macaé 27965-045, RJ, Brazil; caiocheohen@ufrj.br; 7 Programa de Pós-Graduação em Ciências Biológicas, Instituto de Biofísica Carlos Chagas Filho, Universidade Federal do Rio de Janeiro, Rio de Janeiro 21941-590, RJ, Brazil; larissaconstant@biof.ufrj.br (L.E.C.C.); stephanycosta@biof.ufrj.br (S.d.S.C.); diegoallonso@pharma.ufrj.br (D.A.); 8 Departamento de Virologia, Instituto de Microbiologia Paulo de Góes, Universidade Federal do Rio de Janeiro, Rio de Janeiro 21941-590, RJ, Brazil; ptcalil@micro.ufrj.br (P.T.C.); ljcosta@micro.ufrj.br (L.J.d.C.); 9 Laboratory of Morphology and Viral Morphogenesis, Oswaldo Cruz Institute, Oswaldo Cruz Foundation, Rio de Janeiro 21041-250, RJ, Brazil; amanda.tucci@ioc.fiocruz.br (A.R.T.); thamara.oliveira@ioc.fiocruz.br (T.K.F.d.O.); alicerosa@aluno.fiocruz.br (A.d.S.R.); vivian.ferreira@ioc.fiocruz.br (V.N.d.S.F.); julianilo@ufrj.br (J.N.H.L.); mmiranda@ioc.fiocruz.br (M.D.M.); 10 Programa de Pós-Graduação em Biologia Celular e Molecular, Instituto Oswaldo Cruz, Fundação Oswaldo Cruz, Rio de Janeiro 21041-250, RJ, Brazil; 11 Departamento de Química, Universidade Federal da Paraíba, João Pessoa 58033-455, PB, Brazil; mtscotti@ccae.ufpb.br; 12 Departamento de Biotecnologia Farmacêutica, Faculdade de Farmácia, Universidade Federal do Rio de Janeiro, Rio de Janeiro 21941-902, RJ, Brazil; 13 Instituto de Pesquisas de Produtos Naturais, Universidade Federal do Rio de Janeiro, Rio de Janeiro 21941-902, RJ, Brazil

**Keywords:** COVID-19, FRET, pseudotyped virus, PLS regression

## Abstract

The LABEXTRACT plant extract bank, featuring diverse members of the Myrtaceae family from Brazilian hot spot regions, provides a promising avenue for bioprospection. Given the pivotal roles of the Spike protein and 3CL^pro^ and PL^pro^ proteases in SARS-CoV-2 infection, this study delves into the correlations between the Myrtaceae species from the Atlantic Forest and these targets, as well as an antiviral activity through both *in vitro* and *in silico* analyses. The results uncovered notable inhibitory effects, with *Eugenia prasina* and *E. mosenii* standing out, while *E. mosenii* proved to be multitarget, presenting inhibition values above 72% in the three targets analyzed. All extracts inhibited viral replication in Calu-3 cells (EC_50_ was lower than 8.3 µg·mL^−1^). Chemometric analyses, through LC-MS/MS, encompassing prediction models and molecular networking, identified potential active compounds, such as myrtucommulones, described in the literature for their antiviral activity. Docking analyses showed that one undescribed myrtucommulone (m/z 841 [M − H]^−^) had a higher fitness score when interacting with the targets of this study, including ACE2, Spike, PL^pro^ and 3CL^pro^ of SARS-CoV-2. Also, the study concludes that Myrtaceae extracts, particularly from *E. mosenii* and *E. prasina*, exhibit promising inhibitory effects against crucial stages in SARS-CoV-2 infection. Compounds like myrtucommulones emerge as potential anti-SARS-CoV-2 agents, warranting further exploration.

## 1. Introduction

Brazil holds areas of high natural wealth in terms of plant biodiversity, including two out of the thirty-four hotspots spread across the world: Cerrado and the Atlantic Forest. These ecoregions remain highly threatened, subject to rapid urbanization and high levels of resource exploitation [1].

The Myrtaceae family is commonly found in many of these biodiversity hotspots, such as southwestern Australia, as well as in the Brazilian Cerrado and Atlantic Forest [2], where Myrtaceae stands out as one of the most diverse families of woody plants [1]. The LABEXTRACT from the Federal University of Rio de Janeiro is a plant extract bank that holds a representative number of samples, containing species from the Atlantic Forest and the Amazon regions, which represent an outstanding source of potentially pharmacologically active compounds, as well as new chemical entities.

Several studies, by using both *in silico* and *in vitro* approaches, have demonstrated the relevance of the Brazilian flora in tackling SARS-CoV-2 infection and, therefore, the COVID-19 pandemic. Previous studies from our group have shown the capacity of flavonoids isolated from *Siparuna cristata* (Poepp. & Endl.) A. DC. to inhibit SARS-CoV-2 *in vitro* replication [3], as well as the capability of glycosylated phenylethanoid glycosides to act as 3-Quimiotripsin-like protease (3CL^pro^) and Papain-like protease (PL^pro^) inhibitors, of which forsythoside and verbascoside (abundant phenylpropanoids in Brazilian plants) were the most promising multitarget compounds [4]. The potential of *Ampelozizyphus amazonicus* Ducke bark extracts against SARS-CoV-2, which is a plant traditionally used by Brazilian *quilombola* Amazonian riverine communities to treat COVID, has been demonstrated by their capacity to inhibit the interaction between the SARS-CoV-2 Spike protein and angiotensin-converting enzyme 2 (ACE2) receptor and, therefore, reduce viral replication in Calu-3 cells. This effect was attributed to phenolic compounds found in these extracts [5].

SARS-CoV-2’s Spike protein, 3CL^pro^, and PL^pro^ are significant viral targets for developing multitarget compounds to combat the virus [6]. The Spike protein, responsible for viral-host cell fusion, is crucial for the virus’s entry into the host cell [7]. By targeting this protein, the interaction with the host’s ACE2 receptor can be prevented, thereby inhibiting the entry of the virus [8]. 3CL^pro^, a homodimeric protease, is vital for the viral replication process. Its unique structure, comprising three domains and an active site with a catalytic dyad (HIS 41 and CYS 145), makes it an ideal target for antiviral drugs [9,10,11]. PL^pro^, on the other hand, is a monomeric protease with a distinct active site formed by a catalytic triad (CYS 111, HIS 272, and ASP 286). Its enzymatic activity is integral for viral maturation and replication [12,13,14].

The Myrtaceae family has economic importance, as its species are commonly used as condiments, for essential oil extraction, and for wood and fruit production [15]. Moreover, their valuable therapeutic properties, such as antibacterial, antifungal, antiviral, antioxidant, anti-inflammatory, cytotoxic, and antiproliferative activities, have stimulated research efforts to identify bioactive compounds in their extracts. These extracts are known to be rich in terpenes, polyphenols, and other unique acylphloroglucinol compounds named myrtucommulones [2,16,17,18]. These phloroglucinol derivatives can be chemically divided into two main subclasses: oligomeric acylphloroglucinols and phloroglucinol–terpene adducts [2], with a very large structural diversity. Among these compounds, the myrtucommulones are related to various biological activities. Several have shown significant activities against Epstein–Barr virus (EBV), herpes simplex virus (HSV), respiratory syncytial virus (RSV), and human immunodeficiency virus (HIV), and more recently, docking analysis have suggested that they can be effective against SARS-CoV-2 [19,20,21,22].

In this context, the objective of this work was to investigate through chemometric analyses correlations between the bioactive compounds present in extracts of the Myrtaceae family on the entry and replication mechanisms associated with the Spike protein, ACE2, and 3CL^pro^ and PL^pro^ proteases of SARS-CoV-2 in host cells through *in vitro* and *in silico* analysis. The species studied included *Eugenia brasiliensis* Lam., *Eugenia prasina* O. Berg., *Eugenia mosenii* (Kausel) Sobral, *Myrcia strigipes* Mart., and *Myrcia splendens* Sw. DC., all from Atlantic Forest and available in the LABEXTRACT.

## 2. Results and Discussion

### 2.1. Myrtaceae Extracts Inhibit RBD:ACE2 Complex Formation and SARS-CoV-2 Proteases Activity In Vitro

The ability of the extracts to inhibit the formation of RBD:ACE2 complex was assessed by Lumit™ kit (Promega), a bioluminescence-based kit suitable for the trial of potential inhibitory samples [23]. At 250 μg·mL^−1^, only the *E. brasiliensis* (12%) extract was not able to inhibit RBD:ACE2 interaction above 70% (Table 1). The extract of *E. prasina* stood out as the most active, showing an inhibitory rate of 96%, followed by *E. mosenii* extract (84%).

The FRET-based assay was employed to measure SARS-CoV-2 3CL^pro^ and PL^pro^ proteolytic activities by using the fluorescence resonance energy transfer (FRET) approach. This assay has been applied to investigate potential enzyme activity inhibitors from both synthetic and natural sources [24].

As presented in Table 1, only the extract from *M. strigipes* exhibits inhibitory activity below 50% against SARS-CoV-2 r3CL^pro^. Notably, the *E. mosenii* extract displays inhibitory activity exceeding 80%. Regarding activity against SARS-CoV-2 rPL^pro^, the extracts from *E. brasiliensis* and *M. strigipes* demonstrated inhibitory activities below 50%, while *E. mosenii* displayed the highest inhibition value (72%).

### 2.2. Myrtaceae Extracts Inhibit SARS-CoV-2 Spike-Pseudotyped VSV Entry into VERO ACE2 Cells

**Figure 1:**
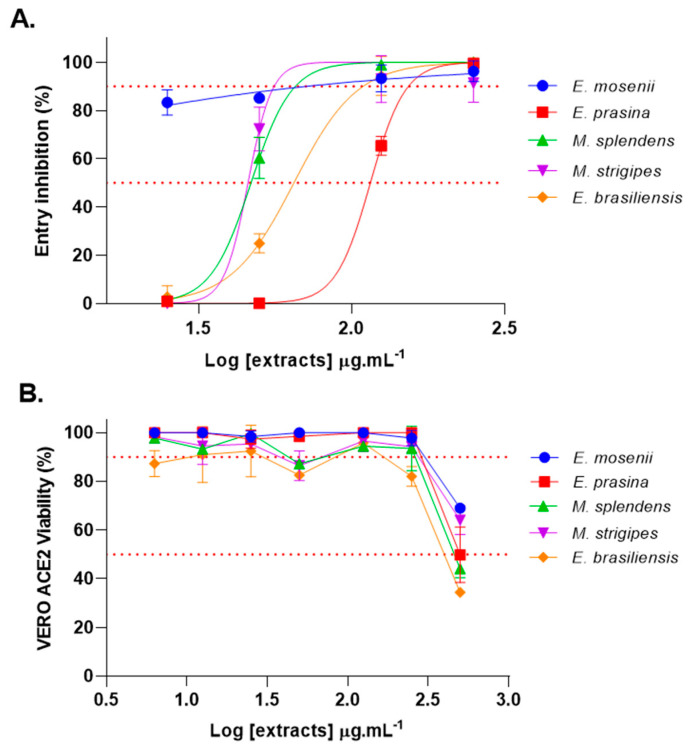
(**A**) Inhibition of Spike-pseudotyped VSV receptor-mediated cell entry after treatment with Myrtaceae extracts (*E. prasina*, *M. splendens*, *M. strigipes*, *E. mosenii*, and *E. brasiliensis*) in four different concentrations (250, 125, 50, and 25 μg·mL^−1^), assessed by luciferase activity assay. Percent neutralization was determined by quantification of total luciferase signal resulting from successful pseudovirus infection, normalized to vehicle control (n = 3). (**B**) Vero E6-ACE2 cells viability after treatment with Myrtaceae extracts in different concentrations (500, 250, 125, 50, 25 and 12.5 μg·mL^−1^) was assessed with an MTT colorimetric test (5 mM, 1× PBS). Total cell viability was determined by quantification of dye metabolization by untreated cells normalized to vehicle control (n = 3). Lines in the graphs mark, respectively, 90% and 50% of inhibition.

Since most Myrtaceae extracts showed good inhibition of the RBD:ACE2 complex formation, a SARS-CoV-2 Spike-pseudotyped VSV that contains in the envelope the SARS-CoV-2 Spike protein and a VSV replication machinery with a luciferase reporter gene was used to measure the potential of Myrtaceae extracts to block SARS-CoV-2 Spike-mediated cell entry. For this assay, Vero E6 cells that overexpress ACE2 (Vero E6/ACE2) were used. When the Spike pseudotyped viruses are incubated with cells containing the ACE2, it is possible for them to recognize the receptor and attach and enter the cells through the binding between the Spike protein and the receptors on the cell surface. It had been previously shown using RBD:ACE2 complex formation assays that Myrtaceae extracts present the ability to inhibit the protein–protein interaction, suggesting that substances in the extracts could act as blockers of virus cell entry. In follow-up neutralization assays, using Spike-pseudotyped VSV, extracts were able to significantly block receptor-mediated cell entry (Figure 1A) at the highest tested concentration (250 μg·mL^−1^). The extract from *E. mosenii* seemed to be the most active, inhibiting cell entry up to 80% even at the lowest tested concentration (25 μg·mL^−1^) and presenting the lowest EC_50_ value (<25 μg·mL^−1^) (Table 2). The extracts only induced cell toxicity in concentrations above 500 μg·mL^−1^ (Figure 1B).

### 2.3. Chemometric Analyses Using Partial Least Squares Regression Prediction Model

To predict the potential active compounds that inhibit all the Spike:ACE2 interactions (Lumit™ kit) and r3CL^pro^ and rPL^pro^ proteolytic activities (FRET), the 15 ethanol extracts from the five species of the Myrtaceae family underwent liquid chromatography coupled to tandem mass spectrometry (LC-MS/MS) analysis using two ionization sources in both positive and negative modes (LC-ESI/APCI-(+/−)-MS/MS). Chemometric analyses were performed alongside binary values of activities in the three target models (excluding SARS-CoV-2 Spike-pseudotyped VSV entry assay). A Partial Least Squares (PLS) regression analysis model was constructed to correlate the LC-MS data with the inhibitory activity of the extracts.

The PLS regression used the LC-MS dataset of the 15 samples as independent variables (X), encompassing a total of 170 parameters (*m*/*z* ions), with the dependent variable (Y) being the inhibition activities against the three target models. This algorithm combines principal component analysis (PCA) and linear regression, seeking latent variables that maximize the correlation between predictors (*m*/*z* ions) and biological activity. Based on the PLS analyses, patterns in the sample distribution were identified, allowing classification into two groups based on average activity values across the three target models: more active samples (≥50% inhibition; active) and less active samples (<50% inhibition; non-active).

All four chemical datasets (ESI+/− and APCI+/−) for each plant extract were modeled, and the data obtained from negative electrospray ionization (ESI) showed the highest explained variance, R² and Q² values, and the lowest Root Mean Square Error (RMSE) in the four constructed models. Therefore, data from this ionization mode were selected to proceed with prediction analyses. At the outset, three models were developed to individually evaluate the Spike:ACE2, r3CL^pro^, and rPL^pro^ targets. Within these models, our goal was to assess the influence of ions obtained through LC-MS/MS analyses in explaining the inhibitory activity response. Consequently, this approach enabled us to identify ions exhibiting the strongest correlation with the target under investigation, thereby providing insights into their potential roles in the conducted experimental assays.

In the investigation model of Spike:ACE2 interaction inhibitors, the explained variance was 99.7% utilizing five factors, which is higher than the values observed in the models for investigating r3CL^pro^ inhibition (81% with six factors) and rPL^pro^ inhibition (85% with four factors). Similar trends were observed in terms of accuracy and reproducibility parameters: R2/Q2 values of 0.95/0.91, 0.77/0.65, and 0.85/0.74 for Spike:ACE2, r3CL^pro^, and rPL^pro^, respectively (Supplementary Figures S1–S3). In all cases, the Root Mean Square Error of Calibration (RMSEC) was below 0.2, and the Root Mean Square Error of Cross-Validation (RMSECV) was under 0.3. These parameters collectively indicate a robust fit for the constructed models.

In the investigation of potential inhibitors for the Spike:ACE2 interaction (Supplementary Figure S1), ten ions with contributions exceeding 0.1 (loadings weights) were identified: *m*/*z* 996.4, 911.5, 841.3, 826.6, 741.6, 661.4, 453.4, 445.1, 439.4, and 431.1. In the other models, ten and seven ions were found with the same contribution threshold: *m*/*z* 841.3, 661.4, 611.1, 555.0, 453.4, 445.1, 439.4, 431.1, 341.3, and 271.1 for r3CL^pro^ (Supplementary Figure S2) and *m*/*z* 841.3, 661.4, 457.4, 453.4, 445.1, 439.4, and 431.1, for rPL^pro^ (Supplementary Figure S3).

**Figure 2:**
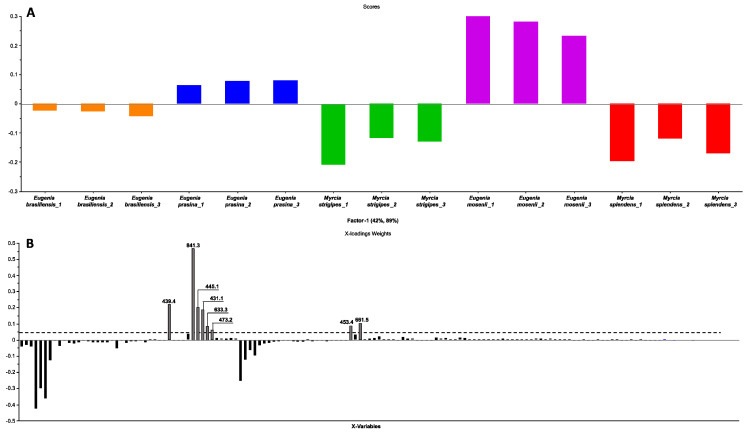
Scores (**A**), X-loadings weight (**B**) for the PLS model based on the LC-MS data obtained in the electrospray ionization in negative mode of the 15 samples of five species of Myrtaceae family, and the inhibition values of the extracts on the RBD:ACE2 interaction and the activity of the r3CL^pro^ and rPL^pro^ enzymes of SARS-CoV-2. The dashed line indicates the cutoff for contributions exceeding 0.05 (loadings weights).

When examining the one-target models that were created, it becomes evident that there are certain ions displaying contributions exceeding the established threshold, and some ions are similar across all three evaluated targets. As a result, the decision was made to develop a fourth model, a multitarget model. This choice was based on the premise that molecules capable of affecting more than one target hold greater promise in the quest for compounds with anti-SARS-CoV-2 potential. To construct this model, the same data matrix and normalization techniques employed in the one-target models were used, with the sole distinction lying in the assessed response. The response (Y) for this model was formulated following this logic: samples exibiting activity in all three targets were categorized as multitarget active (1, Active), whereas samples demonstrating activity in only one or two targets were designated as one-target active (−1, Non-active) (Figure 2A,B).

The multitarget PLS model achieved an explained variance of 98.1% during calibration, employing seven factors and 96.9% during internal validation. The model presented an R² of 0.98 and a Q² of 0.96, indicating a reliable fit. Additionally, it had low RMSE values, with RMSECV (validation error) at 0.15, higher than RMSEC (calibration error) at 0.11. An expected RMSECV > RMSEC relationship confirmed the representativeness of the calibration data and the absence of overfitting in the model.

In the score plot of the PLS model (Figure 2A), the separation between the two distinct groups is evident (active and non-active). The X-loadings weights plot (Figure 2B) reveals the ions that make the most positive contribution in explaining the desired activity, specifically, ions that are found exclusively and/or in greater abundance in extracts with higher activity. This process identified eight ions with *m*/*z* values of [M − H]^−^ 431.1, 439.4, 445.1, 453.4, 473.2, 633.3, 661.5, and 841.3.

### 2.4. Annotated Compounds for the Predicted Ions in the Multitarget Model Using Molecular Networking Analyses

Molecular networks are now widely used in the natural products chemistry area since the creation of the open-source Global Natural Products Social Molecular Networking (GNPS) online platform [25]. This tool allows the visualization of tandem mass spectrometry data (MS^2^) to highlight structural similarities between metabolites of complex matrices and to help in the annotation of the detected metabolites [26].

**Figure 3:**
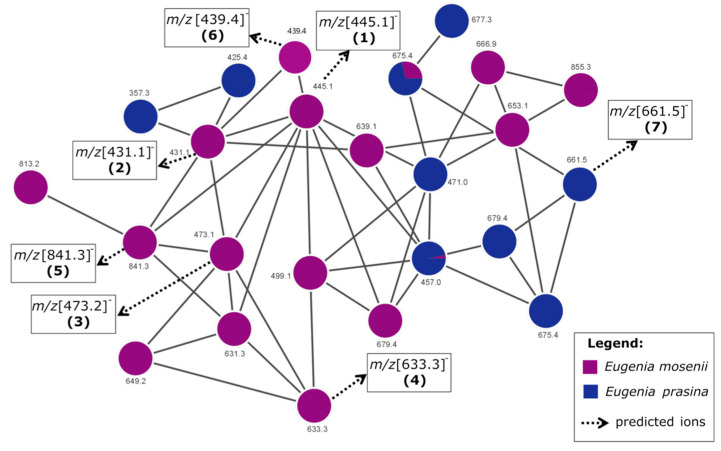
Network 1 showing the oligomeric acylphloroglucinols type compounds, ions that most contribute to the anti-SARS-CoV-2 activity.

The clusters formed can be considered “molecular families” since the compounds should share key chemical characteristics for the node’s connection. The predicted ions in the chemometric analysis formed a network whose compounds did not match the GNPS library search. However, through this concept of molecular networks, they could belong to the same class of compounds. The ions that most contribute to the anti-SARS-CoV-2 activity were grouped into Network 1 (Figure 3).

The molecular networks 1 and 2 (Figure 3 and Supplementary Figure S4) display the nodes of the predicted ions with *m*/*z* [445.1]^−^ (**1**), *m*/*z* [431.1]^−^ (**2**), *m*/*z* [473.2]^−^ (**3**), *m*/*z* [633.3]^−^ (**4**), *m*/*z* [841.3]^−^ (**5**), *m*/*z* [439.4]^−^ (**6**), *m*/*z* [661.5]^−^ (**7**), and *m*/*z* [453.4]^−^ (**8**). It is interesting to note that these ions were more prevalent in *E. mosenii*, which was the most active and multitarget species among the analyzed anti-SARS-CoV-2 targets. However, the species *E. prasina* also presents compounds contained in Networks 1 and 2, which could partially explain the separation of these two species from the others as being the most active in the multitarget PLS model (Figure 2).

**Figure 4:**
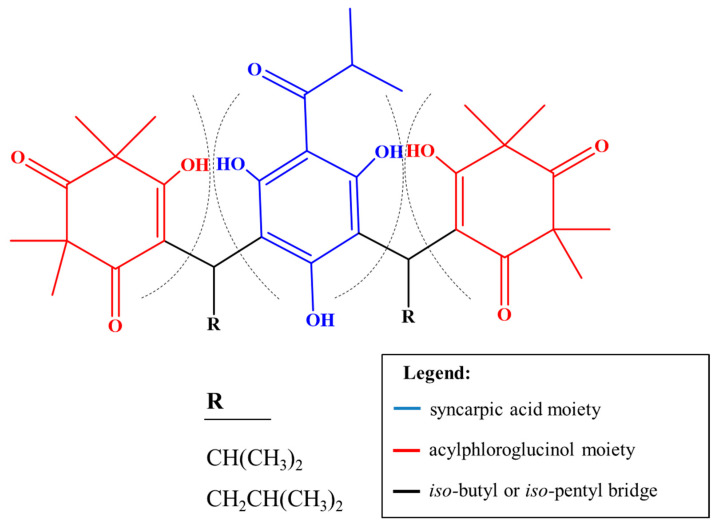
General structure of myrtucommulones and related acylphloroglucinols. Dashed lines denote characteristic fragmentation patterns observed in LC-ESI(-)MS/MS (adapted from [2]).

The parent masses and MS^2^ fragments of these compounds strongly suggest they are myrtucommulones, a class of oligomeric acylphloroglucinols commonly found in the Myrtaceae species (Figure 4). These compounds have attracted the attention due to their diverse molecular architectures that, in general, are composed of an acylphloroglucinol nucleus attached to one or more syncarpic acid residues via an *iso*-butyl or *iso*-pentyl bridge(s) [2,17].

Assignments were made based on the presence of characteristic ions from the portions of syncarpic acid and acylphloroglucinol, resulting from the cleavage of these molecules on both sides of the iso-butyl or iso-pentyl bridge (Figure 4 and Table 3). This mechanism was previously described for *β*-triketone heterodimers linked by an iso-pentyl moiety found in the acetonitrile extract of *Eucalyptus gregsoniana*, a species from the Myrtaceae family [27]. Of the eight predicted ions, two compounds were annotated based on previously described fragmentation patterns [17], and three were proposed as undescribed compounds (Table 3). The remaining ions *m*/*z* [439.4]^−^ and *m*/*z* [661.5]^−^ have the same fragmentation pattern and are present in Network 1 of the myrtucommulone class (Figure 3). The ion at *m*/*z* of [453.4]^−^ exhibits a comparable fragmentation pattern and is found within Network 2 of other acylphloroglucinol derivatives. It displays the mass corresponding to the acylphloroglucinol monomer in the MS/MS spectrum (Supplementary Figure S4).

Compounds **1** (semimyrtucommulone) and **2** (*nor*-semimyrtucommulone) were previously described in *Myrtus communis* and have acylphloglucinol and syncarpic acid residues linked by an iso-butyl bridge [17], as well as compound 3, not yet reported in the literature. These structures are classified as dimeric type myrtucommulones [2].

**Figure 5:**
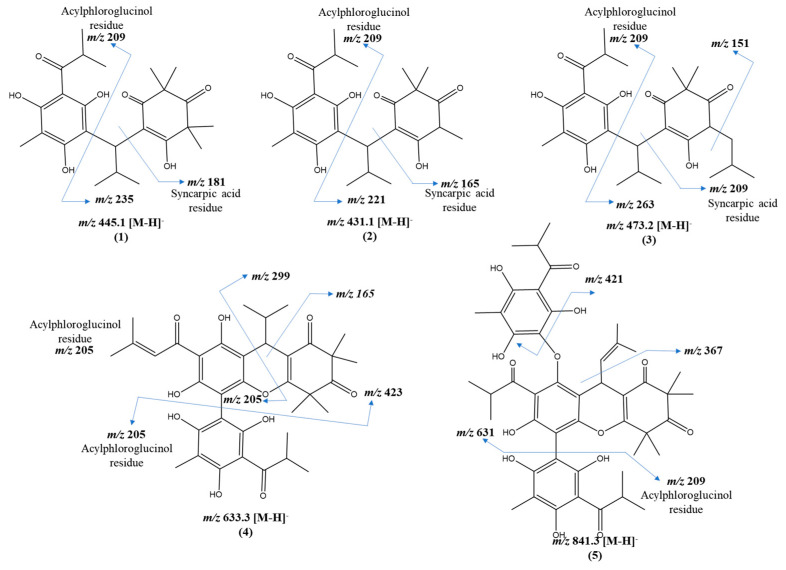
Structure and proposed fragmentation of annotated compounds (**1**–**5**). Compounds **1** and **2** (semimyrtucommulone and *nor*-semi myrtucommulone, respectively) were isolated as racemic mixtures [28,29].

The fragmentations follow the aforementioned patterns (Figure 5), which means that cleavage on one side of the iso-butyl bridge creates the pairs of fragments at *m*/*z* [209]^−^ and *m*/*z* [235]^−^ for compound **1**, *m*/*z* [209]^−^, *m*/*z* [221]^−^ for compound **2**, and *m*/*z* [209]^−^ and *m*/*z* [263]^−^ for compound **3**. The ion at *m*/*z* [209]^−^ is characteristic of the acylphloroglucinol residue [2]. The subsequent cleavage on the other side of the iso-butyl bridge generates the fragments at *m*/*z* [181]^−^, *m*/*z* [165]^−^, and *m*/*z* [209]^−^ for compounds **1**, **2**, and **3**, respectively. These ions are characteristic of the syncarpic acid residue (Supplementary Figures S5–S7).

Compounds **4** and **5** present the same fragmentation pattern but are characteristic of trimeric and tetrameric molecules, respectively, meaning that they contain more than two residues of acylphloroglucinol and/or syncarpic acid (Figure 5, Supplementary Figures S8 and S9). Similarly to compounds **3**, **4** and **5**, several unknown compounds with a similar fragmentation pattern were observed in Network 1, suggesting that new compounds of this class may be present in *E. mosenii* and *E. prasina*.

Phloroglucinols and their derivatives, in particular myrtucommulones, display diverse molecular architectures and a wide range of biological profiles, such as antimicrobial, antiviral, anti-inflammatory, antiproliferative, and cytotoxic properties [2].

The antiviral activity of the hydroalcoholic leaf extracts of *Myrtus communis* L. was investigated by Moradi et al. [19] against herpes simplex virus-1 (HSV-1), who observed an inhibitory effect on the virus both before and after entering the cell. In the review study conducted by Nicoletti et al. [17], myrtucommulones extracted from species of the genus *Kunzea* and *Melaleuca* also showed moderate effects against HSV-1.

Myrtucommulones and related acylphloroglucinols from Myrtaceae were evaluated for their *in vitro* activity against the respiratory syncytial virus (RSV), showing promising effects compared with the ribavirin control. Studies suggested that the antiviral activity of these compounds might be influenced by the side chains of the phloroglucinol moiety [20,21]. Phloroglucinols have also been shown to be effective against other viruses, such as human immunodeficiency virus (HIV), herpes or enterovirus, and preliminary docking analysis data suggest that they can be effective against SARS-CoV-2 [22]. In addition, macrocarpals A-E (phloroglucinol dialdehyde diterpene derivatives) were isolated from *Eucalyptus globulus* Labill. (Myrtaceae) showed pronounced anti-HIV activity [2].

**Figure 6:**
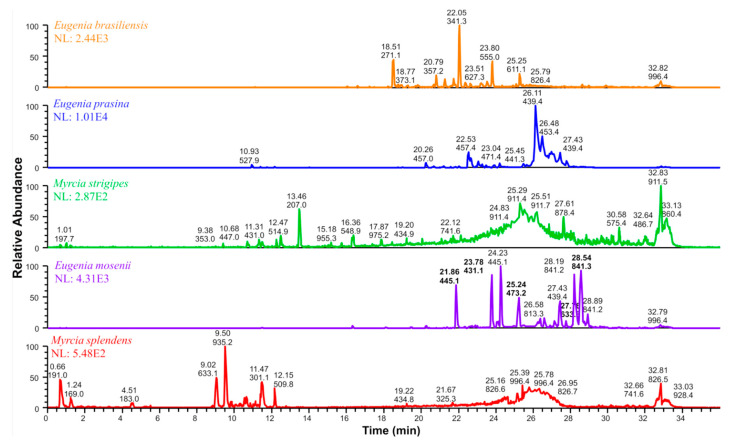
Aligned chromatograms of Myrtaceae ethanol extracts by UHPLC-ESI(-)3D-IT-MS/MS highlighting the predicted ions.

Liquid chromatography coupled with tandem mass spectrometry (UHPLC-MS/MS) analysis of the five Myrtaceae ethanol extracts showed chemical differences in their profiles (Figure 6), evidencing the more significant presence of myrtucommulones in the multitarget extracts (*E. prasina* and *E. mosenii*). Through the Feature Based Molecular Network (FBMN) workflow, other molecular networks were constructed (Supplementary Figure S4). For the unpredicted ions, there was a match in the GNPS library for 2′,6′-dihydroxy-4′-methoxydihydrochalcone (*m*/*z* 271.1 [M − H]^−^) in *E. brasiliensis* (Supplementary Figure S10) [30] and myricitrin for *E. prasina* [31], as well as the presence of quinic and ellagic acids for *M. splendens* [32], confirmed by their fragmentation profiles. In addition, *M. splendens* and *M. strigipes* show chemical similarities in the range of 24 to 28 min, with evidence of the presence of tannins already reported in the literature for species of the Myrtaceae family [32].

The recognition that Myrtaceae extracts act as blockers of the ACE2:RBD interaction in Vero E6/ACE2 cells infected with Spike-pseudotyped VSV and as inhibitors of the SARS-CoV-2 r3CLpro and rPLpro proteolytic activities led us to investigate the Myrtaceae extracts as inhibitors of the SARS-CoV-2 replication in infected Calu-3 cells, a model of human lung epithelial cells (human pneumocytes type II model).

### 2.5. Myrtaceae Extracts Inhibit SARS-CoV-2 Replication in Infected Lung Cells

Despite Vero E6 cells being recognized as an important lineage for SARS-CoV-2 replication [33], they may not adequately mimic the infection event in human cells [34,35]. Therefore, in a second phase, we conducted experimental assays with the human pneumocyte lineage, widely recognized as the most effective model for *in vitro* infection and screening of anti-coronavirus drugs due to its high permissibility to SARS-CoV-2 through a transmembrane serine protease 2 (TMPRSS2)-dependent mechanism viral entry [36,37,38].

**Figure 7:**
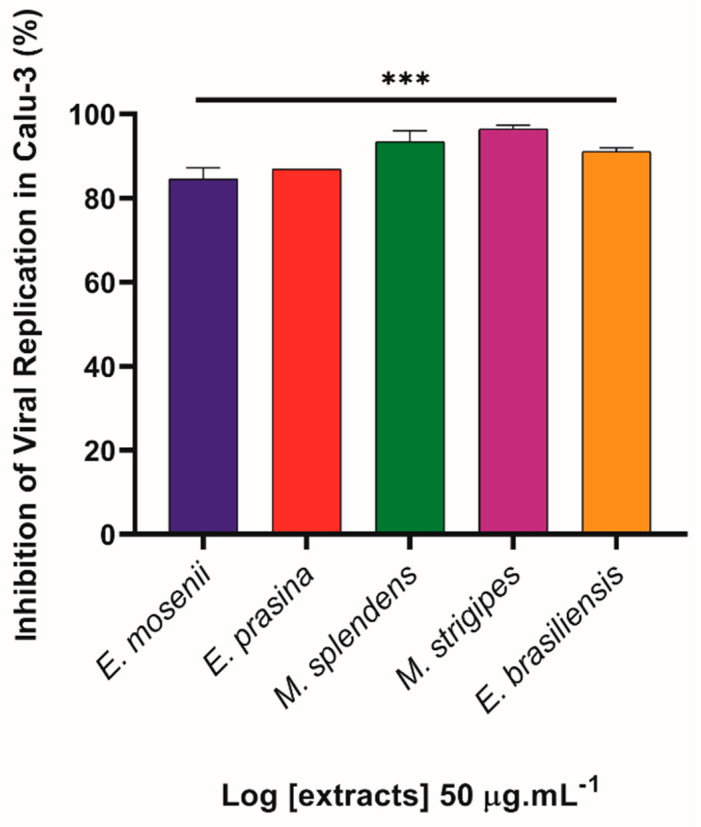
Myrtaceae extracts inhibit virus replication in Calu-3 cells. Infected cells with SARS-CoV-2 at MOI (multiplicity of infection) 0.01 were treated with five different plant extracts at 50 µg·mL^−1^ for 24 h at 37 °C and 5% CO_2_. After this period, the supernatants were harvested, and the virus titles were accessed by plaque-forming units (PFU/mL) assay. The percentage of inhibition was obtained by comparison with the infected/untreated group. *** *p* ≤ 0.001 in relation to infected control (n = 4).

Firstly, Calu-3 cells were infected at a multiplicity of infection (MOI) of 0.01, and the extracts were evaluated for their antiviral activity at a single concentration (50 µg·mL^−1^) for 24 h of treatment, aiming to select the plant species with the best antiviral performance. However, the data indicated that all extracts exhibited high potential as inhibitors of SARS-CoV-2 replication, reducing viral infection by more than 80% (Figure 7).

**Figure 8:**
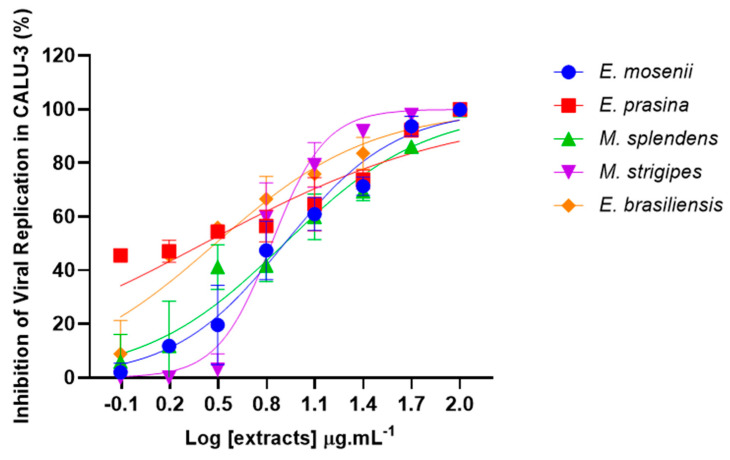
Effect of Myrtaceae species on the SARS-CoV-2 replication in Calu-3 cells. Calu-3 infected with SARS-CoV-2 (MOI: 0.01) for 1 h were treated with different concentrations (0.781, 1.563, 3.125, 6.25, 12.5, 25, 50, and 100 µg·mL^−1^) of the extracts at 37 °C and 5% CO_2_. The supernatants were harvested 24 h after infection for virus titration by a plaque-forming units (PFU/mL) assay. The percentage of inhibition was obtained by a comparison with infected/untreated cells and infected/treated cells. R^2^ values ranged from 0.77 to 0.97 (n = 4).

Based on the initial screening, we further investigated whether the anti-SARS-CoV-2 activity of the studied species would remain effective in Calu-3 cells that were infected and exposed to different extract concentrations (curve: 0.781, 1.563, 3.125, 6.25, 12.5, 25, 50, and 100 µg·mL^−1^) for 24 h. *In vitro* analysis demonstrated that the five Myrtaceae extracts were able to inhibit approximately 100% of SARS-CoV-2 replication when treated with a high tested concentration (100 µg·mL^−1^). Remarkably, it was observed that the high antiviral effect was maintained in treatments until 12.5 µg·mL^−1^ (100–60%) (Figure 8). We highlight that all extracts presented lower EC_50_ values in Calu-3 cells, with a concentration below 8.15 µg·mL^−1^ (Table 4). These data indicate that the concentration required to obtain 50% of maximum inhibition in SARS-CoV-2-Calu-3 cells is lower than in VSV-Vero E6/ACE2 cells.

**Figure 9:**
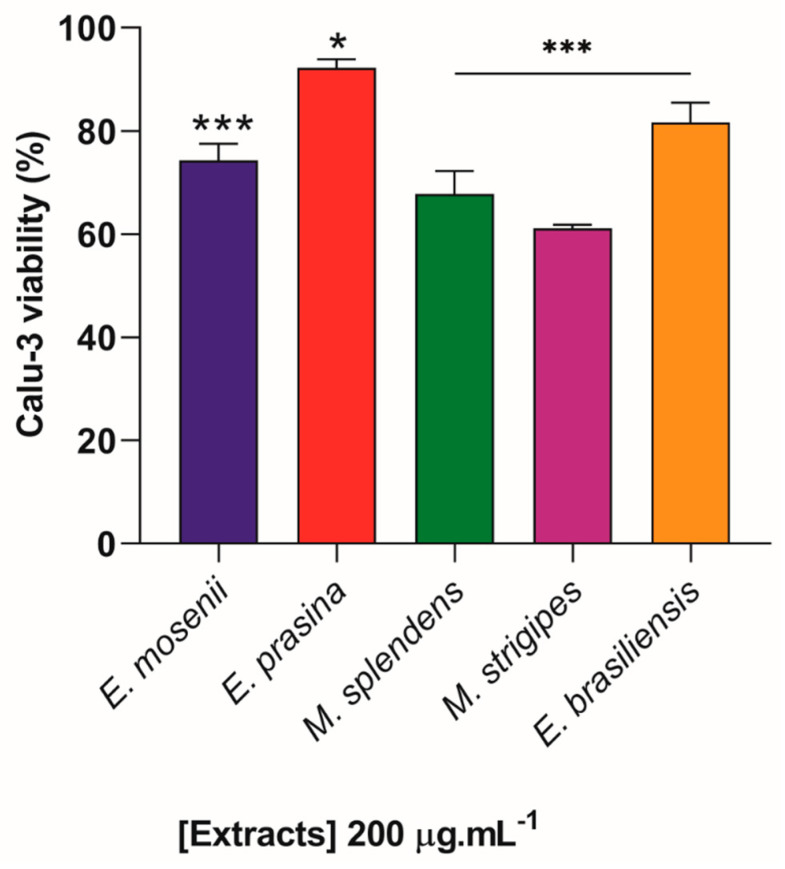
Cytotoxicity of plant extracts in Calu-3 cells. Uninfected cells were exposed to 200 µg·mL^−1^ for 72 h at 37 °C, 5% CO_2_. Cell viability was determined using the LDH assay. * *p* ≤ 0.05 and *** *p* ≤ 0.001 in relation to DMSO-exposed cells (control) (n = 4).

Considering that the extracts were toxic to Vero E6/ACE2 cells only at concentrations above 500 µg·mL^−1^, we aimed to assess the viability of Calu-3, non-infected and treated with the intermediate concentration of 200 μg·mL^−1^ for 72 h, using the colorimetric assay that detects lactate dehydrogenase (LDH) enzyme. Overall, all five plant species showed only low or moderate cytotoxicity. The results demonstrated that the best cell viability indices were observed in treatments with extracts from *E. prasina* (92%), *E. brasiliensis* (82%), and *E. mosenii* (74%), while *M. strigipes* was responsible for causing the greatest cytotoxic effect in a pulmonary cell model (40%) (Figure 9). The concentration used in cytotoxicity assays was up to 24 times higher than the maximum EC_50_ obtained in antiviral assays. The viability data were obtained in comparison with control cells treated with vehicle (DMSO) in the same proportion.

Plant extracts and plant-derived secondary metabolites have broad-spectrum antimicrobial activity and can exhibit properties against SARS-CoV-2 replication [5,39,40,41]. Here, chemometric analyses suggest that myrtucommulones, more prevalent in the multitarget species *E. mosenii* and *E. prasina*, may be the main contributor to the strong inhibitory virus effect exhibited by these extracts. Furthermore, our antiviral assays with Calu-3 cells revealed that other species within this family also demonstrated potent effects against the coronavirus, raising the hypothesis that the action of these biocompounds may occur on other proteases that are also targets of the infection or even that antiviral efficacy may vary in different cell lineages.

Interestingly, studies have revealed that extracts from the species *Scutellaria barbata* D. Don (SBD), belonging to the Lamiaceae family, inhibited the action of the viral proteases CLpro and TMPRSS2, indicating that SBD blocked the entry of SARS-CoV-2 pseudoparticles into the Calu-3 cells by affecting TMPRSS2 activity [42]. Molecular docking analyses revealed that the bioactive compound gingerol, extracted from *Zingiber officinale* (*Zingiberaceae* family), prevents the entry and fusion of coronavirus by blocking the TMPRSS2 receptor [43,44]. Flavonoids such as kaempferol, luteolin, sulforaphane, quercetin, and cryptotanshinone, found in different plant families, also exhibit inhibitory properties on the same serine protease, in addition to acting as anti-inflammatory agents in the treatment of this respiratory disease [45]. In summary, these findings elucidate the several active compounds found in medicinal plants and open ways for future research that encompasses the mechanisms of action of acylphloroglucinol compounds and their immunomodulatory effects in COVID-19, as well as the role of the Myrtaceae family on other therapeutic targets, such as the transmembrane serine protease 2, required for SARS-CoV-2 invasion into pneumocytes cells.

In view of the promising results obtained from the *in vitro* assays and the prediction studies, we decided to perform molecular docking analyses for compounds **1** to **5** to characterize their behavior at the binding sites (3CL^pro^, PL^pro^, and Spike) and to elucidate the existing molecular interactions.

### 2.6. Docking Analysis and Ligand Interactions of Compounds ***1***–***5*** with Spike Protein and ACE2

#### 2.6.1. Molecular Docking of ACE2 and Spike Protein

The comprehensive molecular docking analysis with the SARS-CoV-2 Spike protein and ACE2 has provided us with a deeper understanding of the potential binding affinities of various ligands. The fitness score, a reflection of binding strength, was used as the primary metric for evaluation.

For interactions with the SARS-CoV-2 Spike protein, the ligand compound **3** (*m*/*z* 473 [M − H]^−^) had a score of 19.18, while compound **4** (*m*/*z* 633 [M − H]^−^) achieved 18.24. The ligand compound **5** (*m*/*z* 841 [M − H]^−^) stood out with a score of 20.20, outperforming the other candidates, which prompted us to focus on its interaction analyses. Meanwhile, *nor*-semimyrtucommulone (**2**) and semimyrtucommulone (**1**) had scores of 14.15 and 19.17, respectively (Table 5).

Turning our attention to ACE2, the ligand compound **3** achieved 45.41, and compound **4** displayed a score of 56.37. Remarkably, compound **5** reached a significant score of 67.74, further emphasizing its potential importance in the interaction landscape. The ligands *nor*-semimyrtucommulone (**2**) and semimyrtucommulone (**1**) showed scores of 42.18 and 44.65, respectively.

Collectively, these scores underline the potential interactions and affinities of the ligands with both the Spike protein and ACE2 (Table 5). Our decision to prioritize the interaction analyses for ligand compound **5** was driven by its superior docking scores, suggesting a promising interaction profile.

#### 2.6.2. Interactions of the Ligand Compound **5** with ACE2

Our investigation into the molecular interactions of ACE2 has unveiled additional specific interactions, emphasizing the complexity of ACE2’s molecular associations.

Starting with hydrophobic interactions, the residue 321 PRO displayed an interaction at 3.98 Å. The residue 376 HIS showed an interaction at 3.91 Å. The residue 479 PHE presented a hydrophobic interaction with 3.86 Å. Remarkably, the residue 485 TYR displayed two distinct interactions at distances of 3.39 Å and 3.67 Å, respectively.

In the realm of hydrogen bonds, the residue 322 THR formed a bond with 1.64 Å, an angle of 2.43°, and a dihedral angle of 138.95°, involving atoms 4855 [O3] and 2621 [O3]. The residue 485 TYR demonstrated a bond with 2.67 Å, an angle of 3.28°, and a dihedral angle of 123.42°, connecting atoms 3948 [O3] and 4859 [O3]. Another interesting observation was with residue 489 ARG, which showed two hydrogen bonds: the first with a distance of 3.03 Å, an angle of 3.73°, and a dihedral angle of 129.78° involving atoms 3983 [Ng+] and 4861 [O2]; the second with a distance of 2.00 Å, an angle of 2.96°, and a dihedral angle of 164.71° connecting atoms 3984 [Ng+] and 4861 [O2]. Lastly, the residue 490 TYR established two hydrogen bonds: the first at a distance of 3.19 Å, an angle of 3.77°, and a dihedral angle of 121.26° involving atoms 4862 [O3] and 3996 [O3] and the second with a distance of 2.56 Å, an angle of 3.40°, and a dihedral angle of 150.14° connecting atoms 3996 [O3] and 4853 [O2].

Lastly, our data also highlighted π-cation interactions. Specifically, the residue 489 ARG showcased a π-cation interaction with 4.36 Å and a deviation of 1.98 Å.

#### 2.6.3. Interactions of the Ligand Compound **5** with the Spike Protein

In our in-depth analysis of molecular interactions between the ligand and the SARS-CoV-2 Spike protein, specific interactions have been identified, highlighting the possible intricate association between them.

Amongst the hydrophobic interactions, the Spike protein residue 340 GLU showed an interaction at 3.32 Å. The residue 341 VAL exhibited an interaction at 3.93 Å. The residue 344 ALA displayed a hydrophobic interaction with 3.66 Å. Furthermore, the residue 346A ARG formed an interaction at 3.35 Å. The residue 347 PHE also demonstrated a hydrophobic interaction with 3.48 Å. The residue 354 ASN presented an interaction with 3.57 Å. Impressively, the residue 358 ILE showcased two distinct interactions: the first with 3.86 Å and the second at a shorter distance of 3.04 Å.

Regarding hydrogen bonds, the Spike protein residue 340 GLU formed a bond with 1.57 Å, an angle of 2.53°, and a dihedral angle of 167.25°, involving atoms 58 [O3] and 290 [O.CO2]. The residue 354 ASN established a bond at 2.27 Å, an angle of 3.16°, and a dihedral angle of 149.37°, connecting atoms 407 [Nam] and 49 [O2]. Another significant interaction was observed with the residue 356A LYS displaying a bond distance of 2.29 Å, an angle of 2.73°, and a dihedral angle of 104.76°, involving atoms 428 [N3+] and 48 [O2].

While our comprehensive investigation has elucidated a multitude of interactions between the ligand and both the SARS-CoV-2 Spike protein and ACE2, it is noteworthy to mention that the interactions identified are not among those described in known hotspots. Given the significance of hotspot interactions in biological function and therapeutic targeting, this observation suggests that these proteins may not be the most promising candidates for further molecular dynamic studies. Future work will prioritize ligands and protein targets that exhibit interactions within or near known functional hotspots.

### 2.7. PL^pro^ and 3CL^pro^ Interaction Analysis and Molecular Dynamics

The docking results for PL^pro^ showed that compound **5** (*m*/*z* 841 [M − H]^−^) had the highest score (69.08), followed by compound **3** (*m*/*z* 473 [M − H]^−^) (61.07), *nor*-semimyrtucommulone (**2**) (49.57), semimyrtucommulone (**1**) (48.71), and compound **4** (*m*/*z* 633 [M − H]^−^) (37.19) respectively.

**Figure 10:**
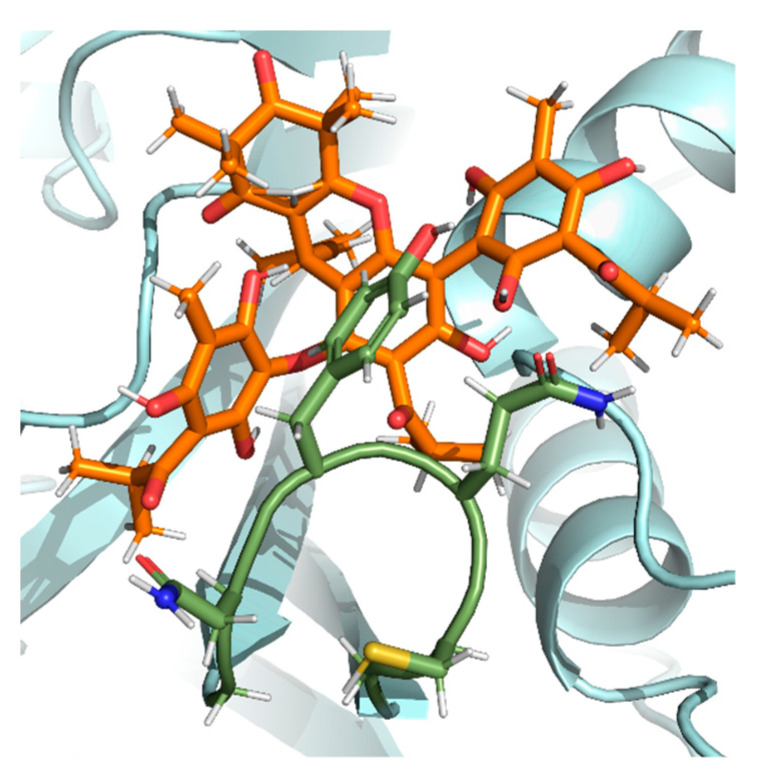
Interaction of the PL^pro^–compound **5** complex in the BL2loop. The protein is colored in pale cyan, the residues of BL2loop are colored in green (residues G266, N267, Y268, Q269, C270, and G271), and the ligand compound **5** is colored in orange (PDBid: 7JRN).

Chemical interaction predictions between PL^pro^ and the molecules were made. Compound **3** made H-bonds with ASP 164 (1.21 Å) and ARG 166 (1.41 Å). Semimyrtucommulone (**1**) h-bonds were predicted with ARG 166 (1.60 Å and 2.68 Å) and TYR 264 (1.70 Å). Compound **4** was predicted to do h-bonds with GLU 167 (1.55 Å). Both compound **5** (Figure 10) and nor-semimyrtucommulone (**2**) had no h-bond predicted.

As seen with PL^pro^, the docking analyses showed that compound **5** also obtained the highest score with 3CL^pro^, equal to 65.01, among the substances analyzed. Compound **4** had the second-best score (59.99), followed by compound 3 (58.43), *nor*-semimyrtucommulone (**2**) (55.55), and semimyrtucommulone (**1**) (50.14), in that order. The evaluation of the receptor–ligand interaction for compound **5** revealed an H-bond between O7 (Supplementary Figure S11) and CYS145 (2.32 Å), representative of the HIS41-CYS145 catalytic dyad [10]. OH29 from the same position forms an H-bond with ARG188 (2.39 Å), and OH23 forms a bond at 2.52 Å with GLN189, located in the S2 region of the active groove [46].

**Figure 11:**
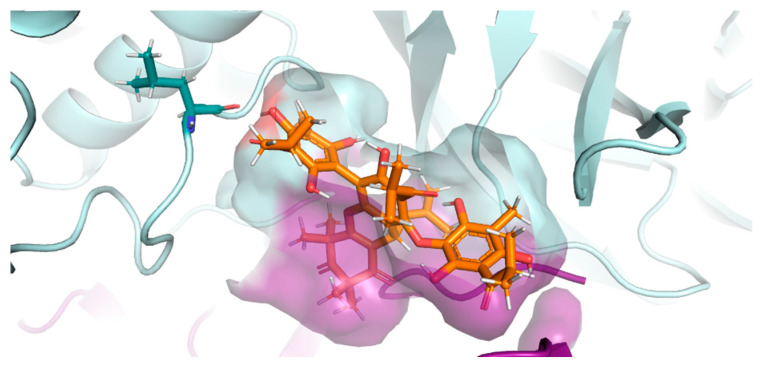
Interaction of the 3CL^pro^–compound **5** complex in the allosteric site. The A-chain of the homodimer is colored in grayish green, and the B-chain residues that interacted in the cavity are colored in magenta. A-Leu287 is represented by dark green sticks. Compound **5** is shown in orange within the alternative interaction groove (PDBid: 6XQT).

Considering that 3CL^pro^ has a described allosteric binding region between the homodimer chains [10], the possible inhibition of the enzyme at this site was also tested. Again, compound **5** stood out from the other compounds, with a score of 63.61. Figure 11 shows the interaction of compound **5** in the region between the protease chains. A chemical interaction was predicted with OH29 of the ligand (Supplementary Figure S11), which formed a 1.71 Å H-bond with Leu287 of the A chain. The ranking of the other compounds continues with compound **4** leading with a 63.37 score, then semimyrtucommulone (**1**) (56.61), then compound **3** (54.02), and lastly the *nor*-semimyrtucommulone (**2**) (53.41).

**Figure 12:**
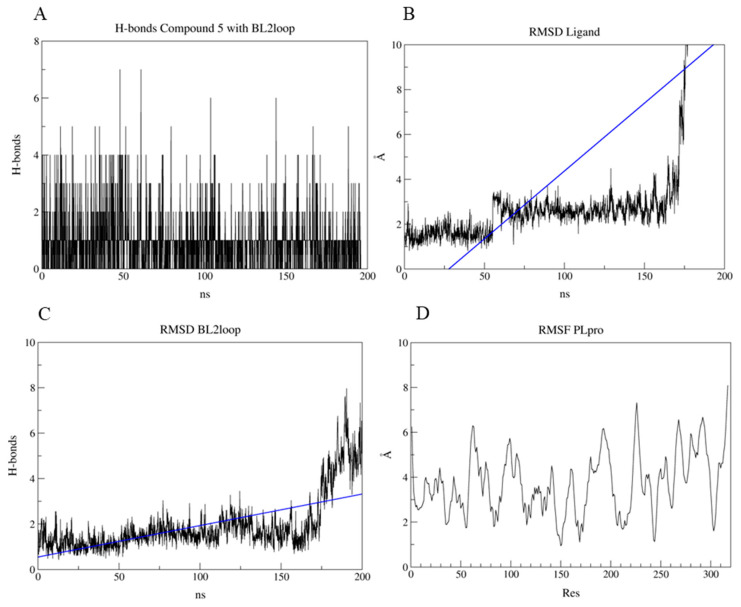
Results of the molecular dynamics of the PL^pro^–compound **5** complex in 200 ns. (**A**) Predicted h-bonds between the BL2loop residues and compound **5**. (**B**) Calculated RMSD of the ligand compound **5** for the duration of the simulation. (**C**) Calculated RMSD of the BL2loop for the duration of the simulation. (**D**) Calculated RMSF for the residues of the PL^pro^.

To further investigate the results obtained, MD were performed, and the results can be viewed in the Supplementary Data (Movies S1–S3). These simulations were used to evaluate the dynamics of the interaction between the PL^pro^–compound **5** (Movie S1) and 3CL^pro^ active site–compound **5** (Movie S2) and 3CL^pro^ allosteric groove–compound **5** (Movie S3) complexes. Our results for PL^pro^ showed that in the first 50 nanoseconds of our simulation, we observed that the average number of hydrogen bonds formed between PL^pro^ and compound **5** was typically around 3. However, during certain moments within these nanoseconds, this number increased, reaching up to 7 hydrogen bonds (Figure 12A). After 50 ns, the average became 2 h-bonds formed until the last 10 ns. In this mark, no h-bonds were predicted (Figure 12A). Ligand RMSD has a 3 staged profile, with an average of 1.8 Å during the first 50 ns, 2.5 Å from 50 ns to approximately 175 ns, and in the last of the 25 ns reaching an RMSD higher than 10 Å (Figure 12B). This high RMSD and the lack of predicted h-bonds at the end of the simulation allude to the exit of the ligand from the binding site. This is demonstrated in the RMSD of the BL2loop, which maintains an average of 1.8 Å until approximately 175 ns when the average climbs to approximately 4.5 Å, showing that this region has moved (Figure 12C). The RMSF graph (Figure 12D) shows how the BL2loop residues (residues 266, 267, 268, 269, 270, 271) highly fluctuated during the simulation (approximately 5 Å, with some residues reaching 6 Å).

**Figure 13:**
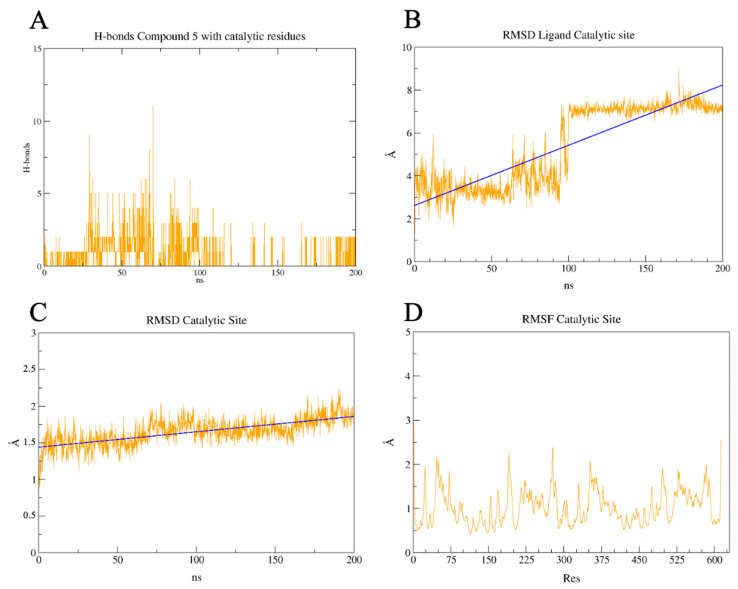
Results of the molecular dynamics of the 3CL^pro^-compound 5 complex in 200 ns. (**A**) Predicted h-bonds between compound **5** and the residues CYS145, HIS41, GLU166, ARG188, and GLN189. (**B**) Calculated RMSD of the ligand compound **5** for the duration of the simulation. (**C**) Calculated RMSD of the catalytic site for the duration of the simulation. (**D**) Calculated RMSF for the residues of the 3CL^pro^.

Furthermore, the MD results for complex 3CL^pro^–compound **5** targeted to the active site of the protease did not show any hydrogen bonds with residues of interest (HIS41 and CYS145). However, an average of 2 h-bond was reported during the 200 ns of simulation, with greater frequency up to 100 ns (Figure 13A). In this period, the residues with the highest occupancy were GLN189 and ARG188, confirming the result obtained with Maestro. Moreover, the ligand RMSD (Figure 13B) showed instability with a plateau starting at 110 ns, maintaining an average of 7 Å. This can be justified due to the larger size of the ligand and the fact that the active site of the protease is shallow [47], so when it reaches this plateau there is a smaller area of the ligand that can remain stable in the interaction with the target. In contrast to the instability of the ligand, the catalytic cavity remained relatively stable during the 200 ns (Figure 13C), maintaining a mean RMSD of 1.5 Å. Our results indicate that although the ligand remained partially in the groove, it did not generate stability in the protein, given the increase in ligand RMSD (Figure 13B) and the increase in variability assessed by RMSF. Figure 13D shows high movement in the region formed by the residues of interest (HIS41, CYS145, GLU166, GLN189, and ARG188).

**Figure 14:**
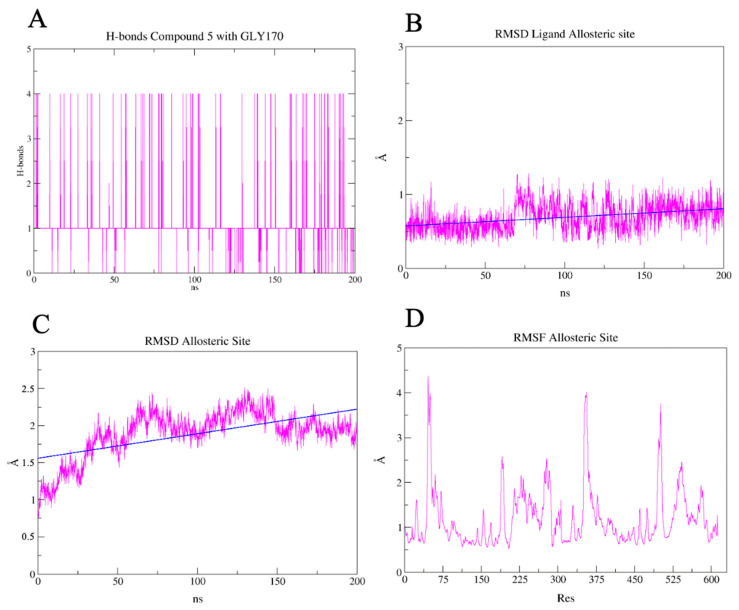
Results of the molecular dynamics of the 3CL^pro^-compound **5** complex in 200 ns. (**A**) Predicted h-bonds between compound **5** and the residues GLY170. (**B**) Calculated RMSD of the ligand compound **5** for the duration of the simulation. (**C**) Calculated RMSD of the allosteric site for the duration of the simulation. (**D**) Calculated RMSF for the residues of the 3CL^pro^.

The results of the simulation in aqueous solution showed greater stability of the ligand in the allosteric site than when compared to the active site. The compound **5** ligand did not show any h-bond with LEU287. However, GLY170 stood out. A 95% occupancy and an average of 4 h-bonds with GLY170 and compound **5** were predicted over the entire simulated time (Figure 14A). In contrast to the ligand RMSD targeted to the active site, the ligand RMSD in the predicted allosteric pocket showed greater stability (Figure 14B), with an average RMSD of less than 1 Å over the 200 ns. While the cavity was more unstable, the RMSD varied between 0.5 Å and 2.5 Å over 200 ns (Figure 14C). One hypothesis is that this is due to greater perturbation of the residues for the ligand to fit into the cavity. This can also be assessed by the flexibility of the residues in the RMSF plot of the protein (Figure 14D).

Moving forward to the analysis of the pharmacokinetic properties, the Lipinski rule of 5 criteria predicts that compound **3**, semimyrtucommulone (**1**), and *nor*-semimyrtucommulone (**2**) are promising drugs. Furthermore, Table 6 shows that their lipophilicity satisfies membrane permeability according to the LogP value. The Caco-2 assessment of the same three compounds indicates satisfactory absorption criteria, which are presented in Supplementary Table S1. While compounds **4** and **5** are metabolized through their properties as inhibitors of cytochromes CYP1A2, CYP2C19, CYP2D6, and CYP3A4.

The high content of Plasma Protein Binding (PPB) restricts the bioavailability of all five compounds. Moreover, all compounds must be capable of crossing the Blood–Brain Barrier (BBB) (Supplementary Table S1). The tested molecules are expected to cause hepatotoxicity in humans (H-HT) and drug-induced liver injury (DILI). Nevertheless, they do not demonstrate genotoxicity, as concluded by the Ames test. The compounds do not have carcinogenic or respiratory toxicity properties (see Table 7), except for semimyrtucommulone **1**, which shows potential for both. In addition, since the *in silico* prediction indicated the presence of a PAINS warning, it is possible that this higher promiscuity in compounds **4** and **5** can interfere with the *in vitro* testing and, therefore, generate false-positive results.

In brief, compound **5** shows greater promise in interacting with ACE2-Spike, PL^pro^, and 3CL^pro^ targets of SARS-CoV-2 based on *in silico* analysis. Nonetheless, its physicochemical features suggest that structural modifications to the molecule may be required. Such changes may also enhance the receptor-ligand complex interaction, rendering compound **5** an encouraging multitarget drug candidate.

## 3. Materials and Methods

### 3.1. Chemicals, Materials and Plant Extracts

*E. brasiliensis* (RB00658589/AL1707) and *M. strigipes* (RB01071480/TROVO526) were collected in the Floresta da Tijuca (State of Rio de Janeiro), *E. mosenii* (RB00776077/AL1901) and *M. splendens* (RB00662950/AL1721) were collected in the Parque Nacional de Itatiaia (State of Rio de Janeiro), and *E. prasina* (RB00831331/PARDOC2233) was collected in Parque Nacional da Serra dos Órgãos (PARNASO—State of Rio de Janeiro). The first code in parentheses refers to the voucher specimen number deposited at Jardim Botanico do Rio de Janeiro herbarium, and the second represents the original reference code in the extract bank. The leaves were dried in a ventilated oven and grounded in a hammer mill. Extracts of the leaves were obtained by maceration with ethanol: water (9:1), filtrated, and the solvent removed by rotary evaporation under reduced pressure. These extracts belong to the Federal University of Rio de Janeiro plant extract bank (LABEXTRACT, Centro de Ciências da Saúde, Universidade Federal do Rio de Janeiro). This work was authorized by the Directing Council of Genetic Heritage (Conselho de Gestão do Patrimônio Genético, CGEN) with bioprospecting purposes by the authorization ACADBDD.

### 3.2. Human Lung Cells Culture

The human lung submucosal gland cells (Calu-3), kindly donated by the Farmanguinhos RPT11M platform, were cultured and maintained in high-glucose medium—Dulbecco’s modified Eagle medium (DMEM, GibcoTM, Waltham, MA, USA) supplemented with 10% fetal bovine serum (FBS, GibcoTM, Waltham, MA, USA), 1% penicillin/streptomycin (GibcoTM, Waltham, MA, USA) and Hepes (GibcoTM, Waltham, MA, USA), and maintained at 37 °C with 5% CO_2_ atmosphere. Calu-3 cells were seeded into 96-well plates (1.5 × 10^4^ cells/well; Jet Biofil, Guangzhou, China) and incubated for 120 h in an incubator and then used for cytotoxicity or antiviral assays.

### 3.3. SARS-CoV-2 Virus

The SARS-CoV-2 B.1 lineage (GenBank#MT710714, SisGen AC58AE2) was maintained in growth in culture flasks (150 cm^2^, Jet Biofil, Guangzhou, China) containing semi-confluent Vero E6 cells for 72 h at 37 °C and 5% CO_2_. The virus supernatant was collected and stored at −80 °C. In all procedures, the SARS-CoV-2 culture was handled at a biosafety level 3 (BSL3) in the multiuser environment according to World Health Organization (WHO) guidelines [48].

### 3.4. SARS-CoV-2 RBD:ACE2 Interaction

The commercial kit Lumit™ SARS-CoV-2 Spike RBD:ACE2 immunoassay (Promega, Madison, WI, USA) was used to measure the inhibition of the RBD and ACE2 interaction following the instructions of the manufacturer and as previously described [5]. For this, 1 mg of each sample was separately solubilized in DMSO (dimethyl sulfoxide—Sigma-Aldrich, St. Louis, MO, USA) to a final concentration of 10 mg·mL^−1^. The solutions were centrifuged (5804 R—Eppendorf) for 5 min at 2500 RPM, and the supernatants were collected. After the reaction, the resulting bioluminescence was recorded in a SpectraMax M5 (Molecular Devices, San Jose, CA, USA) microplate reader at 500 ms of exposure.

### 3.5. SARS-CoV-2 Spike-Pseudotyped VSV Neutralization Assay

SARS-CoV-2 Spike pseudotyped virus was constructed using a human vesicular stomatitis virus (VSV) packaging system. The pseudoviruses were produced in HEK293-T cells, carrying the luciferase gene as a reporter. Briefly, cells were transfected with VSV-G plasmid using Lipofectamine 3000 (Invitrogen, Waltham, MA, USA) reagent following the instructions provided by the manufacturer to produce VSV-Luciferase viruses, which were then used to perform the infection in the cells transfected with original SARS-CoV-2 Spike protein expression plasmid.

The neutralization assay using the SARS-CoV-2 Spike-pseudotyped VSV was performed using Vero E6 cells overexpressing ACE2, seeded in flat bottom 96-well plates in a concentration of 5 × 10⁴ per well (ThermoFisher Scientific, Waltham, MA, USA). The viruses were pre-treated with the extracts in different concentrations (25, 50, 125 and 250 μg·mL^−1^) for 1 h and then added to cells, and the plates were incubated for 24 h at 37 °C and 5% CO_2_. After incubation, luciferase substrate was added to cells (ONE-Glo™ Luciferase Assay System, Promega, Madison, WI, USA) following manufacturer instructions, and after 30 min, luminescence was recorded in a SpectraMax M5 (Molecular Devices, San Jose, CA, USA) microplate reader in 1000 ms of exposure.

All the extracts were resuspended in 100% dimethyl sulfoxide (DMSO) for the *in vitro* tests in a proportion not exceeding 1% (*v*/*v*) for the final concentration and not affecting cell growth.

### 3.6. In Vitro Inhibition of SARS-CoV-2 3CL^pro^ and PL^pro^

Recombinant SARS-CoV-2 3CL^pro^ and PL^pro^ expressed in *E. coli* BL21(DE3)pLysS and BL21(DE3) cells, respectively, were used in a fluorescent resonance energy transfer (FRET) assay using the peptides DABCYL-AVLQ↓SGFRKE-EDANS as substrate for 3CL^pro^ and DABCYL-ALKG↓GKIVE-Glu(EDANS) for PL^pro^. The 3CL^pro^ concentration was fixed at 1.5 μM and the substrate at 50 μM, and the extracts were tested at 100 μM using a high concentration in order to assess the maximum inhibitory effect promoted by each extract. The mixture was incubated in 5 mM NaCl, 20 mM Tris.HCl pH 8.0, and 5 mM DTT for 15 min at 37 °C prior to starting with the substrate. For PL^pro^, a similar protocol was used, with the exception that the enzyme concentration was fixed at 1 μM and the reaction buffer was 150 mM NaCl and 20 mM Tris.HCl pH 8.0, and 5 mM DTT. The emission fluorescence of EDANS was monitored in the following parameters: λexc = 330 nm and λem = 490 nm at 37 °C for 45 min. Fluorescence data (RFU) were converted into substrate cleavage-specific activity using fluorescent conversion factor (FEC) previously calculated based on the EDANS-DABCYL fluorophore pair. Maximum enzyme activity was considered in the situation with vehicle (DMSO), and the values were used to calculate the enzyme inhibition by the compounds.

### 3.7. Cytotoxicity Assays

i. MTT assay: Vero E6 cells (overexpressing ACE2 receptor, ATCC, USA) were cultivated in DMEM High Glucose (Sigma-Aldrich, St. Louis, MO, USA) supplemented with 10% fetal bovine serum and 1% of the antibiotics penicillin and streptomycin (ThermoFisher Scientific, Waltham, MA, USA). Cells were seeded in 96-well plates (1.5 × 10⁴ cell/well) and incubated with the extracts in different concentrations (6.25 to 500 µg·mL^−1^) for 1 h. Then, the viability was measured by adding a 5 mg·mL−1 MTT (3-(4,5-dimethylthiazol-2-yl)-2,5-diphenyltetrazolium bromide) solution (Sigma-Aldrich, St. Louis, MO, USA) in 1× PBS to the cell monolayers for 4 h at 37 °C and 5% CO_2_. Then, 100% dimethyl sulfoxide (DMSO, Sigma-Aldrich, St. Louis, MO, USA) was added, and plates were read in a spectrophotometer at 540 nm.

ii. LDH assay: Calu-3 (1.5 × 10^4^ cells/well) was maintained in a 96-well plate (Jet Biofil, Guangzhou, China) with *E. mosenii*, *E. prasina*, *M. splendens*, *M. strigipes* and *E. brasiliensis*, at a concentration of 200 µg·mL^−1^ for 72 h at 37 °C, 5% CO_2_. The supernatants were collected to evaluate cell viability using the CytoTox 96^®^ Non-Radioactive colorimetric kit (Promega, Madison, USA) according to the manufacturer’s instructions. Lysis solution 1× (Promega, Madison, USA) was used to lyse Calu-3 cells, and purified lactate dehydrogenase (LDH) was used as a positive control. The reaction product was analyzed at 490 nm using a 96-well plate reader (Loccus, São Paulo, Brazil). All the extracts were resuspended in 100% DMSO for the *in vitro* tests. The DMSO final concentrations do not exceed 1% (*v*/*v*) in the experiments, thereby not affecting cell growth.

### 3.8. LC-MS/MS Analysis

Ultra-high-performance liquid chromatography coupled with tandem mass spectrometry (UHPLC-MS/MS) analysis was performed using a UHPLC DionexTM UltiMateTM 3000 system, coupled with an LCQ Fleet (ThermoFisher Scientific, Waltham, MA, USA) consisting of an oven, a solvent degasser, an ultra-high-pressure pump, an autosampler, a diode array detector, and a column temperature manager. An ACQUITY UPLC^®^ BEH C18 reversed-phase column (2.1 × 100 mm, 1.7 µm, Waters, Milford, MA, USA) was used at a flow rate of 0.45 mL·min^−1^. The column temperature was kept at 40 °C, and the mobile phases were 0.1% formic acid in water for A and acetonitrile for B. The gradient elution mode was as follows: 5% B in 0–5 min, 5–100% in 5–25 min, 100% in 25–30 min, 100–5% in 30–31 min, and 5% B in 31–36 min.

The mass spectrometer (MS), equipped with an electrospray ionization (ESI) and atmospheric pressure chemical ionization (APCI) sources and an ion trap analyzer (with 1.000 of resolution), was operated in positive- and negative-ion modes. High-purity nitrogen (N2) was used as the sheath gas (20 arbitrary units) and auxiliary gas (10 arbitrary units). High-purity helium (He) was used as the collision gas. Full scan data acquisition (mass range: *m*/*z* 100–1000) and data-dependent acquisition (topN = 3) were performed. The normalized collision energy of the collision-induced dissociation (CID) cell was set at 35 eV. All analyses were carried out for three different preparation samples of each of the five species of the Myrtaceae family.

### 3.9. Data Processing, Statistical Analysis and Molecular Networking

Graphs of biological data were generated using the GraphPad Prism 8.0 software. The results RBD:ACE2 interaction and r3CL^pro^ and rPL^pro^ inhibition were determined using linear regression. For SARS-CoV-2 Spike-pseudotyped VSV neutralization assay, the EC_50_ values were determined with the nonlinear regression of log (inhibitor) vs. normalized response. Values correspond to the best curve generated based on R^2^ values ≥ 0.9. All experiments were realized with three technical replicates.

The data obtained from LC-MS analyses were converted to the mzML format using the parameters: subset, msLevel 1-2 and polarity positive or negative, as well as Peak Picking, vendor algorithm (msLevel 1-2) in the Proteowizard—MSconvert version 3.02 tool. Then, the data were processed in MZmine v.2.53. The mass detection varied according to the intensity and quality of the signals obtained in each analysis method (ESI or APCI [+/−]). The mass chromatogram construction and wavelet deconvolution were carried out using the ADAP Chromatogram Builder algorithm. Isotopes were eliminated through the Isotopic Peaks Grouper, with the most intense isotope selected as the representative, and alignment was performed using the join aligner. The table list was saved to an ASCII text file.

Process chemical data and biological data were then exported to UnscramblerX software version 10.4. The study aimed to predict potential active compounds inhibiting Spike:ACE2 interaction and proteolytic activities of r3CLpro and rPLpro. Fifteen ethanol extracts from Myrtaceae family species underwent LC-MS/MS analysis in positive and negative modes. Chemometric analyses were performed, and a Partial Least Squares Regression model correlated LC-MS data with inhibitory activities. The PLS regression identified sample patterns, classifying them into two groups based on average activity values: active (≥50% inhibition) and non-active (<50% inhibition) samples.

The MZmine results were uploaded to the open-source Global Natural Products Social (GNPS) online platform, along with the open-source converted files (.mzML) and metadata table. These files were used to build the molecular networks and to dereplicate the present compounds. Along with GNPS annotation, a custom database was also used. The parameters used were those recommended in the GNPS documentation for unity resolution data. The GNPS-generated data were downloaded and processed in Cytoscape 3.9.1 to obtain the results of the molecular networking analysis.

### 3.10. Anti-SARS-CoV-2 Assay in Calu-3 Cells

Monolayers of Calu-3 cells were infected with SARS-CoV-2 at a multiplicity of infection (MOI) of 0.01 for 1 h at 37 °C and 5% CO_2_. The supernatants were removed, and the cells were treated with a concentration curve (0.781, 1.563, 3.125, 6.25, 12.5, 25, 50 and 100 µg·mL^−1^) of Myrtaceae extracts for 24 h. Then, the supernatant was collected and used for the virus titration assay. The concentration of the extract required to obtain 50% of maximum effective virus inhibition (EC_50_) was assessed compared with control cells (infected and untreated cells) [49,50].

### 3.11. Virus Titration

A semiconfluent culture of Vero E6 cells (1 × 10^4^ cells/well) in 96-well plates (Jet Biofil, Guangzhou, China) was infected with serial dilutions (1:200–1:25,600) of supernatants containing SARS-CoV-2 for 1 h at 37 °C and 5% CO_2_. After that, fresh semisolid medium containing 2.4% carboxymethylcellulose (CMC [DMEM-High glucose 10×, 2.4% carboxymethylcellulose, 2% FBS, 1% penicillin and streptomycin, and 4.4% sodium bicarbonate]) was added, and the culture was maintained in an incubator for 72 h. Then, cells were fixed with 4% formalin for 3 h at room temperature and stained with 0.04% crystal violet for 1 h [50]. For the titration control, uninfected cells followed the same steps described above. The virus titers were determined by plaque-forming unit assay (PFU/mL).

### 3.12. Statistical Analysis

Cytotoxicity and antiviral assays on Calu-3 cells were performed with four technical replicates per assay. The generated data were analyzed using GraphPad Prism 10.0 software (GraphPad Software, La Jolla, CA, USA). Differences between the compounds were analyzed using one-way ANOVA with post-testing of Dunnett, and the EC_50_ values were determined using nonlinear regression of Log(inhibitor) vs. normalized response. The equations used to fit the best curve were generated based on R^2^ values 0.81 to 0.92.

### 3.13. Assessment of Pharmacokinetic Characteristics

ADMETlab 2.0 [51] was used to predict the following: pharmacokinetic properties of the molecules of molecular weight (MW), number of hydrogen bond acceptors (nHA), number of hydrogen bond donors (nHD), number of rotatable bonds (nRot), topological polar surface area (TPSA), logarithm of the n-octanol/water distribution coefficient (LogP), pan assay interference compounds (PAINS), prediction of intestinal cellular absorption (Caco-2 permeability), substrate capability prediction (Pgp-sub) or inhibitor (Pgp-inh) of P-glycoprotein, human intestinal absorption (HIA), penetration of blood–brain barrier (BBB), fraction unbound in plasms (Fu), prediction if the molecules were substrate or inhibitors of isozymes CYP, human hepatotoxicity (H-HT), drug-induced liver injury (DILI), Ames test for mutagenicity, carcinogenicity, and respiratory toxicity.

### 3.14. Molecular Docking Analysis

Docking between SARS-CoV-2’s viral proteins—PL^pro^ (Pdbid: 7JRN), 3CL^pro^ homodimer (Pdbid: 6XQT), Spike’s Receptor Binding Domain (RBD) [52] portion and Angiotensin-converting enzyme 2 (ACE2) (PDBid: 7A97)—and the substances semimyrtucommulone, *nor*-semimyrtucommulone, compound **3** (*m*/*z* 473 [M − H]^−^), compound **4** (*m*/*z* 633 [M − H]^−^) and compound **5** (*m*/*z* 841 [M − H]^−^) was performed using GOLD suite with GoldScore score function.

For PL^pro^, the binding site was defined with a centre in the coordinates of the original ligand GRL0617 present in the crystal with radius of 10 Å. The radius simulation for 3CL^pro^ was established with 10 Å from HIS 41 in target docking and 35 Å for blind docking, which in the last case, includes the union of the two chains. For the RBD, a blind docking was realized, with the simulation radius of 70 Å with centre in LEU 455. Finally, docking with ACE2 followed the same methodology described for Spike. However, in the latter case, the radius of 80 A was determined from GLN598 establishing the simulation region.

The interaction analysis between amino acid residues and substances was performed using Maestro (Schrödinger Release 2023-3: Maestro, Schrödinger, LLC, New York, NY, USA, 2023) and Protein-Ligand Interaction Profiler.

### 3.15. Molecular Dynamics

Molecular Dynamics (MD) was performed using the AMBER 2022 and AmberTools2022 [53] software packages. For both PL^pro^ (Pdbid: 7JRN) and 3CL^pro^ (Pdbid: 6XQT), the protein file was protonated to pH 8.0 using an H++ web server (http://newbiophysics.cs.vt.edu/H++/, accessed on 13 October 2023) [54,55,56]. Ligand preparation was performed using the Antechamber tool.

The PL^pro^ complex preparation was performed using the MCPB.py tool [57] and GAMESS-US [58] to properly simulate the zinc atom present in the protein and its effects on protein motility.

Force fields used were ff19SB, gaff2, and OPC for waters. Solvation was made in a truncated Octahedron box with a 12.0 Å distance from the box edge. Salt molarity was calculated using the method described by Machado [59] to neutralize the system and be at 0.15 M.

Relaxation was made with 1000 cycles of Steepest descent. Temperature was set to 310 K. Final simulations were for 200 ns with MonteCarlo Barostat. SHAKE was used for restraining hydrogen bonds. Time step was defined to 0.001.

## 4. Conclusions

The study’s findings indicate that among the five Myrtaceae species examined, two (*E. prasina* and *E. mosenii*) demonstrated noteworthy potential as multitarget agents against SARS-CoV-2 targets (Spike:ACE2, PLpro, and 3CLpro). Moreover, all five species of the Myrtaceae family exhibited high inhibitory capacity against SARS-CoV-2 B.1 lineage replication in the human lung epithelial cells model. Notably, ions identified as myrtucommulones were correlated with the observed activity. In addition, the established workflow employs molecular network databases and predictive chemometric models through tandem mass spectrometry to accelerate the dereplication of molecules before subsequent compound isolation. This abbreviates the lengthy process of bioprospecting using bio-guided fractionation. The comprehensive investigation seamlessly integrates biological and chemical analyses, significantly advancing our understanding of the antiviral properties of Myrtaceae. This study represents the first documentation of such activity for these compound types, encouraging further isolation efforts and testing on the models employed.

## Figures and Tables

**Table 1:** Myrtaceae extracts and their effect on the entry and replication mechanisms associated with the Spike protein (250 μg·mL^−1^) in host cells and inhibition of 3CL^pro^ and PL^pro^ proteases (100 μg·mL^−1^) of SARS-CoV-2.

	Inhibition (%)		
Extracts	Spike:ACE2	3CL^pro^	PL^pro^
*Eugenia brasiliensis*	12	69	34
*Myrcia splendens*	81	58	52
*Eugenia mosenii*	84	100	72
*Myrcia strigipes*	79	46	43
*Eugenia prasina*	96	53	57

**Table 2:** EC_50_ values for the five Myrtaceae extracts in Spike-pseudotyped VSV assay.

	*E. mosenii*	*E. prasina*	*M. splendens*	*M. strigipes*	*E. brasiliensis*
EC_50_ (μg·mL^−1^)	<25	115	47	45	65

**Table 3:** LC-MS/MS data of annotated and proposed compounds (undescribed) in the molecular network (MS/MS spectra in Supplementary Figures S5–S9).

Compound	Rt (min)	Molecular Formula	[M − H]^−^ (*m*/*z*)	MS/MS (MS^2^)	Proposed/Annotated Compound	Relative Content (%)	Ref.
Network 1							
**1**	21.86	C_25_H_34_O_7_	445.1	235, 209, 181	semimyrtucommulone	*E. mosenii* (6.2)	[17,28]
**2**	23.78	C_24_H_32_O_7_	431.1	221, 209, 165, 151	*nor*-semimyrtucommulone	*E. mosenii* (7.9)	[17,29]
**3**	25.24	C_27_H_38_O_7_	473.2	263, 209, 151	undescribed	*E. mosenii* (4.2)	-
**4**	27.76	C_36_H_42_O_1_	633.3	423, 219, 205, 209, 165	undescribed	*E. mosenii* (0.9)	-
**5**	28.54	C_47_H_54_O_1_	841.3	631, 577, 421, 367	undescribed	*E. mosenii* (26)	-

**Table 4:** Myrtaceae extract EC_50_ values.

	*E. mosenii*	*E. prasina*	*M. splendens*	*M. strigipes*	*E. brasiliensis*
EC_50_ (µg·mL^−1^)	8.14 ± 0.6	2.57 ± 0.5	8.15 ± 0.7	6.75 ± 0.4	3.03 ± 0.3

**Table 5:** Fitness of top-ranked individuals from GOLD docking.

Ligand Name	ACE2 (Fitness Score)	Spike (Fitness Score)
compound **3** (*m*/*z* 473 [M − H]^−^)	45.41	19.18
compound **4** (*m*/*z* 633 [M − H]^−^)	56.37	18.24
compound **5** (*m*/*z* 841 [M − H]^−^)	67.74	20.2
*nor*-semimyrtucommulone **2**	42.18	14.15
semimyrtucommulone **1**	44.65	19.17

**Table 6:** *In silico* prediction of physicochemical properties of compounds **1**–**5**.

Compounds	MW (Da)	nHA	nHD	TPSA (Å)	nRot	LogP
compound **3** (*m*/*z* 473 [M − H]^−^)	474.26	7	3	128.97	7	4.706
compound **4** (*m*/*z* 633 [M − H]^−^)	634.28	10	5	178.66	6	7.036
compound **5** (*m*/*z* 841 [M − H]^−^)	842.35	14	7	245.42	10	8.359
*nor*-semimyrtucommulone **2**	432.21	7	3	128.97	5	3.845
semimyrtucommulone **1**	446.23	7	3	128.97	5	4.04

MW: Molecular Weight; nHA: number of hydrogen bond acceptors; nHD: number of hydrogen bond donors; TPSA: topological polar surface area; nRot: number of rotatable bonds; LogP: logarithm of the n-octanol/water distribution coefficient.

**Table 7:** Toxicity prediction. Interpret the colors so that green means the substance is not toxic, yellow means it may be toxic, and red means toxic.

Compounds	H-HT	DILI	AMES	Carc.	R.T.	PAINS
compound **3**						
compound **4**						
compound **5**						
nor-semimyrtucommulone **2**						
semimyrtucommulone **1**						

H-HT: human hepatotoxicity; DILI: drug-induced liver injury; Ames: Ames toxicity; Carc.: carcinogenicity; R.T.: respiratory toxicity; PAINS: pan-assay interference. The green color is the probability of the compound meeting that prediction (values from 0 to 0.3); yellow represents medium probability (values >0.3 to 0.6) and values greater than 0.6 to 1 are represented in red.

## Data Availability

Data are contained within the article.

## Supplementary Materials

The following supporting information can be downloaded at https://www.mdpi.com/article/10.3390/ph17040436/s1, Figure S1: Scores (A), X-loadings weight (B) for the PLS model based on the LC-MS data obtained in the electrospray ionization in negative mode of the 15 samples of five species of Myrtaceae family, and the inhibition values of the extracts on the RBD:ACE2 interaction of SARS-CoV-2. The dashed line indicates the cutoff for contributions exceeding 0.1 (loadings weights); Figure S2: Scores (A), X-loadings weight (B) for the PLS model based on the LC-MS data obtained in the electrospray ionization in negative mode of the 15 samples of five species of Myrtaceae family, and the inhibition values of the extracts on the activity of the r3CL^pro^ enzyme of SARS-CoV-2. The dashed line indicates the cutoff for contributions exceeding 0.1 (loadings weights); Figure S3: Scores (A), X-loadings weight (B) for the PLS model based on the LC-MS data obtained in the electrospray ionization in negative mode of the 15 samples of five species of Myrtaceae family, and the inhibition values of the extracts on the activity of the rPL^pro^ enzyme of SARS-CoV-2. The dashed line indicates the cutoff for contributions exceeding 0.1 (loadings weights); Figure S4: Feature Based Molecular Network (FBMN) of Myrtaceae extracts; Figure S5: MS/MS spectrum of compound **1** at *m*/*z* 445.1 [M − H]^−^ (semimyrtucommulone); Figure S6: MS/MS spectrum of compound **2** at *m*/*z* 431.1 [M − H]^−^ (*nor*-semimyrtucommulone); Figure S7. MS/MS spectrum of proposed compound **3** at *m*/*z* 473.2 [M − H]^−^; Figure S8. MS/MS spectrum of proposed compound **4** at *m*/*z* 633.3 [M − H]^−^; Figure S9. MS/MS spectrum of proposed compound **5** at *m*/*z* 841.3 [M − H]^−^; Figure S10. Molecular network of chalcone and chalcone-derivative compounds and MS/MS spectrum of *m*/*z* 271.1 [M − H]^−^ (2′6′-dihydroxy-4′-methoxy dihydrochalcone); Figure S11. 2D structure of compound **5** (*m*/*z* 841 [M − H]^−^) with atomic positions; Table S1—Description of the pharmacokinetic properties of the compounds **3**, **4**, **5**, nor-semimyrtucommulone (**2**), and semimyrtucommulone (**1**); Movie S1—Molecular dynamics results of the complex PL^pro^ with compound **5**. The protein structure is shown in gray, the BL2loop is shown in yellow, and compound 5 is shown in blue. The file is available in https://doi.org/10.5281/zenodo.10257486 (accessed on 4 December 2023); Movie S2—Molecular dynamics results of the complex 3CLpro active site with compound 5. The protein structure is shown in gray, and compound 5 is shown in orange. The file is available at https://doi.org/10.5281/zenodo.10257448 (accessed on 4 December 2023); Movie S3—Molecular dynamics results of the complex 3CLpro allosteric groove with compound 5. The protein structure is shown in gray, and the compound 5 is shown in magenta. The file is available at https://doi.org/10.5281/zenodo.10257460 (accessed on 4 December 2023).

## References

[1] Wagner M.D.A., Bogoni J.A., Fiaschi P. (2022). Myrtaceae Richness and Distribution across the Atlantic Forest Domain Are Constrained by Geoclimatic Variables. Plant Ecol..

[2] Celaj O., Durán A.G., Cennamo P., Scognamiglio M., Fiorentino A., Esposito A., D’Abrosca B. (2021). Phloroglucinols from Myrtaceae: Attractive Targets for Structural Characterization, Biological Properties and Synthetic Procedures. Phytochem. Rev..

[3] Leal C.M., Leitão S.G., Sausset R., Mendonça S.C., Nascimento P.H.A., De Araujo R., Cheohen C.F., Esteves M.E.A., Leal Da Silva M., Gondim T.S. (2021). Flavonoids from *Siparuna cristata* as Potential Inhibitors of SARS-CoV-2 Replication. Rev. Bras. Farmacogn..

[4] Cheohen C.F.D.A.R., Esteves M.E.A., Da Fonseca T.S., Leal C.M., Assis F.D.L.F., Campos M.F., Rebelo R.S., Allonso D., Leitão G.G., Da Silva M.L. (2023). In Silico Screening of Phenylethanoid Glycosides, a Class of Pharmacologically Active Compounds as Natural Inhibitors of SARS-CoV-2 Proteases. Comput. Struct. Biotechnol. J..

[5] Campos M.F., Mendonça S.C., Peñaloza E.M.C., De Oliveira B.A.C., Rosa A.S., Leitão G.G., Tucci A.R., Ferreira V.N.S., Oliveira T.K.F., Miranda M.D. (2023). Anti-SARS-CoV-2 Activity of *Ampelozizyphus amazonicus* (Saracura-Mirá): Focus on the Modulation of the Spike-ACE2 Interaction by Chemically Characterized Bark Extracts by LC-DAD-APCI-MS/MS. Molecules.

[6] Kaul R., Paul P., Kumar S., Büsselberg D., Dwivedi V.D., Chaari A. (2021). Promising Antiviral Activities of Natural Flavonoids against SARS-CoV-2 Targets: Systematic Review. Int. J. Mol. Sci..

[7] Jackson C.B., Farzan M., Chen B., Choe H. (2022). Mechanisms of SARS-CoV-2 Entry into Cells. Nat. Rev. Mol. Cell Biol..

[8] Scialo F., Daniele A., Amato F., Pastore L., Matera M.G., Cazzola M., Castaldo G., Bianco A. (2020). ACE2: The Major Cell Entry Receptor for SARS-CoV-2. Lung.

[9] Salian V.S., Wright J.A., Vedell P.T., Nair S., Li C., Kandimalla M., Tang X., Carmona Porquera E.M., Kalari K.R., Kandimalla K.K. (2021). COVID-19 Transmission, Current Treatment, and Future Therapeutic Strategies. Mol. Pharm..

[10] Zhang L., Lin D., Sun X., Curth U., Drosten C., Sauerhering L., Becker S., Rox K., Hilgenfeld R. (2020). Crystal Structure of SARS-CoV-2 Main Protease Provides a Basis for Design of Improved α-Ketoamide Inhibitors. Science.

[11] Khan R.J., Jha R.K., Amera G.M., Jain M., Singh E., Pathak A., Singh R.P., Muthukumaran J., Singh A.K. (2021). Targeting SARS-CoV-2: A Systematic Drug Repurposing Approach to Identify Promising Inhibitors against 3C-like Proteinase and 2′-O-Ribose Methyltransferase. J. Biomol. Struct. Dyn..

[12] Bosken Y.K., Cholko T., Lou Y.-C., Wu K.-P., Chang C.A. (2020). Insights Into Dynamics of Inhibitor and Ubiquitin-Like Protein Binding in SARS-CoV-2 Papain-Like Protease. Front. Mol. Biosci..

[13] Gao X., Qin B., Chen P., Zhu K., Hou P., Wojdyla J.A., Wang M., Cui S. (2021). Crystal Structure of SARS-CoV-2 Papain-like Protease. Acta Pharm. Sin. B.

[14] Osipiuk J., Azizi S.-A., Dvorkin S., Endres M., Jedrzejczak R., Jones K.A., Kang S., Kathayat R.S., Kim Y., Lisnyak V.G. (2021). Structure of Papain-like Protease from SARS-CoV-2 and Its Complexes with Non-Covalent Inhibitors. Nat. Commun..

[15] Santos L.S., Alves Filho E.G., Ribeiro P.R.V., Zocolo G.J., Silva S.M., De Lucena E.M.P., Alves R.E., De Brito E.S. (2020). Chemotaxonomic Evaluation of Different Species from the Myrtaceae Family by UPLC-qToF/MS-MS Coupled to Supervised Classification Based on Genus. Biochem. Syst. Ecol..

[16] Alipour G., Dashti S., Hosseinzadeh H. (2014). Review of Pharmacological Effects of *Myrtus communis* L. and Its Active Constituents: *Myrtus communis* L. and Its Active Constituents. Phytother. Res..

[17] Nicoletti R., Salvatore M., Ferranti P., Andolfi A. (2018). Structures and Bioactive Properties of Myrtucommulones and Related Acylphloroglucinols from Myrtaceae. Molecules.

[18] Knauthe A., Mittag S., Bloch L., Albring K.F., Schmidt M., Werz O., Huber O. (2022). Hyperforin and Myrtucommulone Derivatives Act as Natural Modulators of Wnt/β-Catenin Signaling in HCT116 Colon Cancer Cells. Int. J. Mol. Sci..

[19] Moradi M.T., Karimi A., Rafieian M., Kheiri S., Saedi M. (2011). The Inhibitory Effects of Myrtle (*Myrtus communis*) Extract on Herpes Simplex Virus-1 Replication in Baby Hamster Kidney Cells. J. Shahrekord Univ. Med. Sci..

[20] Chen M., Chen L.-F., Li M.-M., Li N.-P., Cao J.-Q., Wang Y., Li Y.-L., Wang L., Ye W.-C. (2017). Myrtucomvalones A–C, Three Unusual Triketone–Sesquiterpene Adducts from the Leaves of *Myrtus communis* ‘Variegata’. RSC Adv..

[21] Liu J., Song J.-G., Su J.-C., Huang X.-J., Ye W.-C., Wang Y. (2018). Tomentodione E, a New *Sec*-Pentyl Syncarpic Acid-Based Meroterpenoid from the Leaves of *Rhodomyrtus tomentosa*. J. Asian Nat. Prod. Res..

[22] Peron G., López A.M., Cabada-Aquirre P., Garay Buenrosto K.D., Ostos Mendoza K.C., Mahady G.B., Seidel V., Sytar O., Koirala N., Gurung R. (2023). Antiviral and Antibacterial Properties of Phloroglucinols: A Review on Naturally Occurring and (Semi)Synthetic Derivatives with Potential Therapeutic Interest. Crit. Rev. Biotechnol..

[23] Alves J., Engel L., De Vasconcelos Cabral R., Rodrigues E.L., De Jesus Ribeiro L., Higa L.M., Da Costa Ferreira Júnior O., Castiñeiras T.M.P.P., De Carvalho Leitão I., Tanuri A. (2021). A Bioluminescent and Homogeneous SARS-CoV-2 Spike RBD and hACE2 Interaction Assay for Antiviral Screening and Monitoring Patient Neutralizing Antibody Levels. Sci. Rep..

[24] Hariono M., Rollando R., Yoga I., Harjono A., Suryodanindro A., Yanuar M., Gonzaga T., Parabang Z., Hariyono P., Febriansah R. (2021). Bioguided Fractionation of Local Plants against Matrix Metalloproteinase9 and Its Cytotoxicity against Breast Cancer Cell Models: *In Silico* and *In Vitro* Study (Part II). Molecules.

[25] Wang M., Carver J.J., Phelan V.V., Sanchez L.M., Garg N., Peng Y., Nguyen D.D., Watrous J., Kapono C.A., Luzzatto-Knaan T. (2016). Sharing and Community Curation of Mass Spectrometry Data with Global Natural Products Social Molecular Networking. Nat. Biotechnol..

[26] Afoullouss S., Balsam A., Allcock A.L., Thomas O.P. (2022). Optimization of LC-MS2 Data Acquisition Parameters for Molecular Networking Applied to Marine Natural Products. Metabolites.

[27] Goodger J.Q.D., Seneratne S.L., Nicolle D., Woodrow I.E. (2016). Foliar Essential Oil Glands of *Eucalyptus* Subgenus *Eucalyptus* (Myrtaceae) Are a Rich Source of Flavonoids and Related Non-Volatile Constituents. PLoS ONE.

[28] Appendino G., Bianchi F., Minassi A., Sterner O., Ballero M., Gibbons S. (2002). Oligomeric Acylphloroglucinols from Myrtle (*Myrtus communis*). J. Nat. Prod..

[29] Hans M., Charpentier M., Huch V., Jauch J., Bruhn T., Bringmann G., Quandt D. (2015). Stereoisomeric Composition of Natural Myrtucommulone A. J. Nat. Prod..

[30] Zaki M.A., Nanayakkara N.P.D., Hetta M.H., Jacob M.R., Khan S.I., Mohammed R., Ibrahim M.A., Samoylenko V., Coleman C., Fronczek F.R. (2016). Bioactive Formylated Flavonoids from *Eugenia rigida*: Isolation, Synthesis, and X-Ray Crystallography. J. Nat. Prod..

[31] de Oliveira F.M.G., Romão W., Kuster R.M. (2018). Identification of Phenolic Compounds in *Eugenia uniflora* Leaves by FTICR MS in Association with Different Ionization Sources. Anal. Methods.

[32] Yoshida T., Amakura Y., Yoshimura M. (2010). Structural Features and Biological Properties of Ellagitannins in Some Plant Families of the Order Myrtales. Int. J. Mol. Sci..

[33] Ogando N.S., Dalebout T.J., Zevenhoven-Dobbe J.C., Limpens R.W.A.L., Van Der Meer Y., Caly L., Druce J., De Vries J.J.C., Kikkert M., Bárcena M. (2020). SARS-Coronavirus-2 Replication in Vero E6 Cells: Replication Kinetics, Rapid Adaptation and Cytopathology. J. Gen. Virol..

[34] Mellott D.M., Tseng C.-T., Drelich A., Fajtová P., Chenna B.C., Kostomiris D.H., Hsu J., Zhu J., Taylor Z.W., Kocurek K.I. (2021). A Clinical-Stage Cysteine Protease Inhibitor Blocks SARS-CoV-2 Infection of Human and Monkey Cells. ACS Chem. Biol..

[35] Mautner L., Hoyos M., Dangel A., Berger C., Ehrhardt A., Baiker A. (2022). Replication Kinetics and Infectivity of SARS-CoV-2 Variants of Concern in Common Cell Culture Models. Virol. J..

[36] White J.M., Schiffer J.T., Bender Ignacio R.A., Xu S., Kainov D., Ianevski A., Aittokallio T., Frieman M., Olinger G.G., Polyak S.J. (2021). Drug Combinations as a First Line of Defense against Coronaviruses and Other Emerging Viruses. mBio.

[37] Kumar S., Sarma P., Kaur H., Prajapat M., Bhattacharyya A., Avti P., Sehkhar N., Kaur H., Bansal S., Mahendiratta S. (2021). Clinically Relevant Cell Culture Models and Their Significance in Isolation, Pathogenesis, Vaccine Development, Repurposing and Screening of New Drugs for SARS-CoV-2: A Systematic Review. Tissue Cell.

[38] Mahoney M., Damalanka V.C., Tartell M.A., Chung D.H., Lourenco A.L., Pwee D., Mayer Bridwell A.E., Hoffmann M., Voss J., Karmakar P. (2021). A Novel Class of TMPRSS2 Inhibitors Potently Block SARS-CoV-2 and MERS-CoV Viral Entry and Protect Human Epithelial Lung Cells. Proc. Natl. Acad. Sci. USA.

[39] Mukherjee P.K., Efferth T., Das B., Kar A., Ghosh S., Singha S., Debnath P., Sharma N., Bhardwaj P.K., Haldar P.K. (2022). Role of Medicinal Plants in Inhibiting SARS-CoV-2 and in the Management of Post-COVID-19 Complications. Phytomedicine.

[40] Jamal Q.M.S. (2022). Antiviral Potential of Plants against COVID-19 during Outbreaks—An Update. Int. J. Mol. Sci..

[41] Gupta S., Singh V., Varadwaj P.K., Chakravartty N., Katta A.V.S.K.M., Lekkala S.P., Thomas G., Narasimhan S., Reddy A.R., Reddy Lachagari V.B. (2022). Secondary Metabolites from Spice and Herbs as Potential Multitarget Inhibitors of SARS-CoV-2 Proteins. J. Biomol. Struct. Dyn..

[42] Huang S.-T., Chen Y., Chang W.-C., Chen H.-F., Lai H.-C., Lin Y.-C., Wang W.-J., Wang Y.-C., Yang C.-S., Wang S.-C. (2021). *Scutellaria barbata* D. Don Inhibits the Main Proteases (M^pro^ and TMPRSS2) of Severe Acute Respiratory Syndrome Coronavirus 2 (SARS-CoV-2) Infection. Viruses.

[43] Tallei T.E., Tumilaar S.G., Niode N.J., Fatimawali, Kepel B.J., Idroes R., Effendi Y., Sakib S.A., Emran T.B. (2020). Potential of Plant Bioactive Compounds as SARS-CoV-2 Main Protease (Mpro) and Spike (S) Glycoprotein Inhibitors: A Molecular Docking Study. Scientifica.

[44] Llivisaca-Contreras S.A., Naranjo-Morán J., Pino-Acosta A., Pieters L., Vanden Berghe W., Manzano P., Vargas-Pérez J., León-Tamariz F., Cevallos-Cevallos J.M. (2021). Plants and Natural Products with Activity against Various Types of Coronaviruses: A Review with Focus on SARS-CoV-2. Molecules.

[45] Fornari Laurindo L., Taynara Marton L., Minniti G., Dogani Rodrigues V., Buzinaro Suzuki R., Maria Cavallari Strozze Catharin V., Joshi R.K., Barbalho S.M. (2023). Exploring the Impact of Herbal Therapies on COVID-19 and Influenza: Investigating Novel Delivery Mechanisms for Emerging Interventions. Biologics.

[46] Kumar Y., Singh H., Patel C.N. (2020). *In Silico* Prediction of Potential Inhibitors for the Main Protease of SARS-CoV-2 Using Molecular Docking and Dynamics Simulation Based Drug-Repurposing. J. Infect. Public Health.

[47] Ullrich S., Nitsche C. (2020). The SARS-CoV-2 Main Protease as Drug Target. Bioorg. Med. Chem. Lett..

[48] WHO—World Health Organization Coronavirus Dashboard. https://covid19.who.int.

[49] Tucci A.R., Da Rosa R.M., Rosa A.S., Augusto Chaves O., Ferreira V.N.S., Oliveira T.K.F., Coutinho Souza D.D., Borba N.R.R., Dornelles L., Rocha N.S. (2023). Antiviral Effect of 5′-Arylchalcogeno-3-Aminothymidine Derivatives in SARS-CoV-2 Infection. Molecules.

[50] Caleffi G.S., Rosa A.S., De Souza L.G., Avelar J.L.S., Nascimento S.M.R., De Almeida V.M., Tucci A.R., Ferreira V.N., Da Silva A.J.M., Santos-Filho O.A. (2023). Aurones: A Promising Scaffold to Inhibit SARS-CoV-2 Replication. J. Nat. Prod..

[51] Xiong G., Wu Z., Yi J., Fu L., Yang Z., Hsieh C., Yin M., Zeng X., Wu C., Lu A. (2021). ADMETlab 2.0: An Integrated Online Platform for Accurate and Comprehensive Predictions of ADMET Properties. Nucleic Acids Res..

[52] da-Costa-Rodrigues B., Cheohen C., Sciammarella F., Pierre-Bonetti-Pozzobon A., Ribeiro L., Nepomuceno-Silva J.L., Medeiros M., Mury F., Monteiro-de-Barros C., Lazoski C. (2022). SARS-CoV-2 Spatiotemporal Genomic and Molecular Analysis of the First Wave of the COVID-19 Pandemic in Macaé, the Brazilian Capital of Oil. Int. J. Mol. Sci..

[53] Case D.A., Aktulga H.M., Belfon K., Cerutti D.S., Cisneros G.A., Cruzeiro V.W.D., Forouzesh N., Giese T.J., Götz A.W., Gohlke H. (2023). AmberTools. J. Chem. Inf. Model..

[54] Anandakrishnan R., Aguilar B., Onufriev A.V. (2012). H++ 3.0: Automating pK Prediction and the Preparation of Biomolecular Structures for Atomistic Molecular Modeling and Simulations. Nucleic Acids Res..

[55] Gordon J.C., Myers J.B., Folta T., Shoja V., Heath L.S., Onufriev A. (2005). H++: A Server for Estimating pKas and Adding Missing Hydrogens to Macromolecules. Nucleic Acids Res..

[56] Myers J., Grothaus G., Narayanan S., Onufriev A. (2006). A Simple Clustering Algorithm Can Be Accurate Enough for Use in Calculations of p*K*s in Macromolecules. Proteins Struct. Funct. Bioinform..

[57] Li P., Merz K.M. (2016). MCPB.Py: A Python Based Metal Center Parameter Builder. J. Chem. Inf. Model..

[58] Barca G.M.J., Bertoni C., Carrington L., Datta D., De Silva N., Deustua J.E., Fedorov D.G., Gour J.R., Gunina A.O., Guidez E. (2020). Recent Developments in the General Atomic and Molecular Electronic Structure System. J. Chem. Phys..

[59] Machado M.R., Pantano S. (2020). Split the Charge Difference in Two! A Rule of Thumb for Adding Proper Amounts of Ions in MD Simulations. J. Chem. Theory Comput..

