# Supplementary material for: Myrtucommulones and Related Acylphloroglucinols from Myrtaceae as a Promising Source of Multitarget SARS-CoV-2 Cycle Inhibitors
Source: Pharmaceuticals (Basel). 2024 Mar 28;17(4):436. doi: 10.3390/ph17040436 (PMC11054083; doi:10.3390/ph17040436)
Supplement: Supplementary file 1 [file pharmaceuticals-17-00436-s001.zip › pharmaceuticals-2935544-supplementary.pdf]

Supplementary material

Myrtucommulones and Related Acylphloroglucinols from Myrtaceae as a Promising Source of Multitarget SARS-CoV-2 Cycle Inhibitors

Simony Carvalho Mendonça [1,†], Brendo Araujo Gomes [1,2,†], Mariana Freire Campos [1,2], Thamirys Silva da Fonseca [3], Maria Eduarda Alves Esteves [4], Bruce Veiga Andriolo [5], Caio Felipe de Araujo Ribas Cheohen [6], Larissa Esteves Carvalho Constant [7], Stephany da Silva Costa [7], Pedro Telles Calil [8], Amanda Resende Tucci [9,10], Thamara Kelcya Fonseca de Oliveira [9,10], Alice dos Santos Rosa [9,10], Vivian Neuza dos Santos Ferreira [9], Julia Nilo Henrique Lima [9], Milene Dias Miranda [9,10], Luciana Jesus da Costa [8], Manuela Leal da Silva [4,5,6], Marcus Tullius Scotti [11], Diego Allonso [7,12], Gilda Guimarães Leitão [13,*] and Suzana Guimarães Leitão [1,2,3,*]

1 Departamento de Produtos Naturais e Alimentos, Faculdade de Farmácia, Universidade Federal do Rio de Janeiro, Rio de Janeiro 21941-902, RJ, Brazil; sy2802@ufrj.br (S.C.M.); brendoo.bc@ufrj.br (B.A.G.); camposmariana@biof.ufrj.br (M.F.C.)

2 Programa de Pós-Graduação em Biotecnologia Vegetal e Bioprocessos, Centro de Ciências da Saúde, Universidade Federal do Rio de Janeiro, Rio de Janeiro 21941-902, RJ, Brazil

3 Programa de Pós-Graduação em Ciências Farmacêuticas, Faculdade de Farmácia, Universidade Federal do Rio de Janeiro, Rio de Janeiro 21941-902, RJ, Brazil; thamirysfonseca@ufrj.br

4 Programa de Pós-Graduação em Biologia Computacional e Sistemas, Instituto Oswaldo Cruz, Rio de Janeiro 21040-900, RJ, Brazil; mariaesteves@aluno.fiocruz.br (M.E.A.E.); manuela@macae.ufrj.br (M.L.d.S.)

5 Programa de Pós-Graduação em Biotecnologia, Instituto Nacional de Metrologia, Qualidade e Tecnologia, Duque de Caxias 25250-020, RJ, Brazil; bruceandriolo@ufrj.br

6 Programa de Pós-Graduação Multicêntrico em Ciências Fisiológicas, Centro de Ciências da Saúde, Instituto de Biodiversidade e Sustentabilidade NUPEM, Universidade Federal do Rio de Janeiro, Macaé 27965-045, RJ, Brazil; caiocheohen@ufrj.br

7 Programa de Pós-Graduação em Ciências Biológicas, Instituto de Biofísica Carlos Chagas Filho, Universidade Federal do Rio de Janeiro, Rio de Janeiro 21941-590, RJ, Brazil; larissaconstant@biof.ufrj.br (L.E.C.C.); stephanycosta@biof.ufrj.br (S.d.S.C.); diegoallonso@pharma.ufrj.br (D.A.)

8 Departamento de Virologia, Instituto de Microbiologia Paulo de Góes, Universidade Federal do Rio de Janeiro, Rio de Janeiro 21941-590, RJ, Brazil; ptcalil@micro.ufrj.br (P.T.C.); ljcosta@micro.ufrj.br (L.J.d.C.)

9 Laboratory of Morphology and Viral Morphogenesis, Oswaldo Cruz Institute, Oswaldo Cruz Foundation, Rio de Janeiro 21041-250, RJ, Brazil;

amanda.tucci@ioc.fiocruz.br (A.R.T.); thamara.oliveira@ioc.fiocruz.br (T.K.F.d.O.); alicerosa@aluno.fiocruz.br (A.d.S.R.); vivian.ferreira@ioc.fiocruz.br (V.N.d.S.F.); julianilo@ufrj.br (J.N.H.L.); mmiranda@ioc.fiocruz.br (M.D.M.)

[10] Programa de Pós-Graduação em Biologia Celular e Molecular, Instituto Oswaldo Cruz, Fundação Oswaldo Cruz, Rio de Janeiro 21041-250, RJ, Brazil

[11] Departamento de Química, Universidade Federal da Paraíba, João Pessoa 58033-455, PB, Brazil; mtscotti@ccae.ufpb.br

[12] Departamento de Biotecnologia Farmacêutica, Faculdade de Farmácia, Universidade Federal do Rio de Janeiro, Rio de Janeiro 21941-902, RJ, Brazil

[13] Instituto de Pesquisas de Produtos Naturais, Universidade Federal do Rio de Janeiro, Rio de Janeiro 21941-902, RJ, Brazil

* Correspondence: ggleitao@ippn.ufrj.br (G.G.L.); sgleitao@pharma.ufrj.br (S.G.L.); Tel.: +55-21-3938-6513 (G.G.L.); +55-21-3938-6414 (S.G.L.)

† These authors contributed equally to this work.

Figure S1. Scores (A), X-loadings weight (B) for the PLS model based on the LC-MS data obtained in the electrospray ionization in negative mode of the 15 samples of five species of Myrtaceae family, and the inhibition values of the extracts on the RBD:ACE2 interaction of SARS-CoV-2. The dashed line indicates the cutoff for contributions exceeding 0.1 (loadings weights).

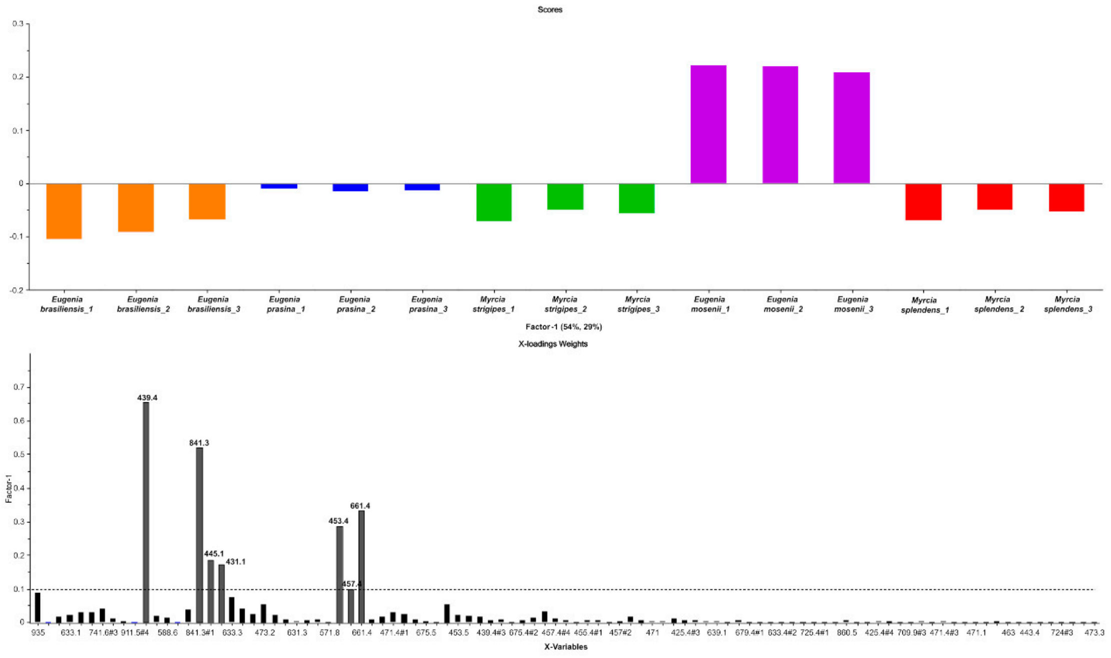

Figure S2. Scores (A), X-loadings weight (B) for the PLS model based on the LC-MS data obtained in the electrospray ionization in negative mode of the 15 samples of five species of Myrtaceae family, and the inhibition values of the extracts on the activity of the r3CLpro enzyme of SARS-CoV-2. The dashed line indicates the cutoff for contributions exceeding 0.1 (loadings weights).

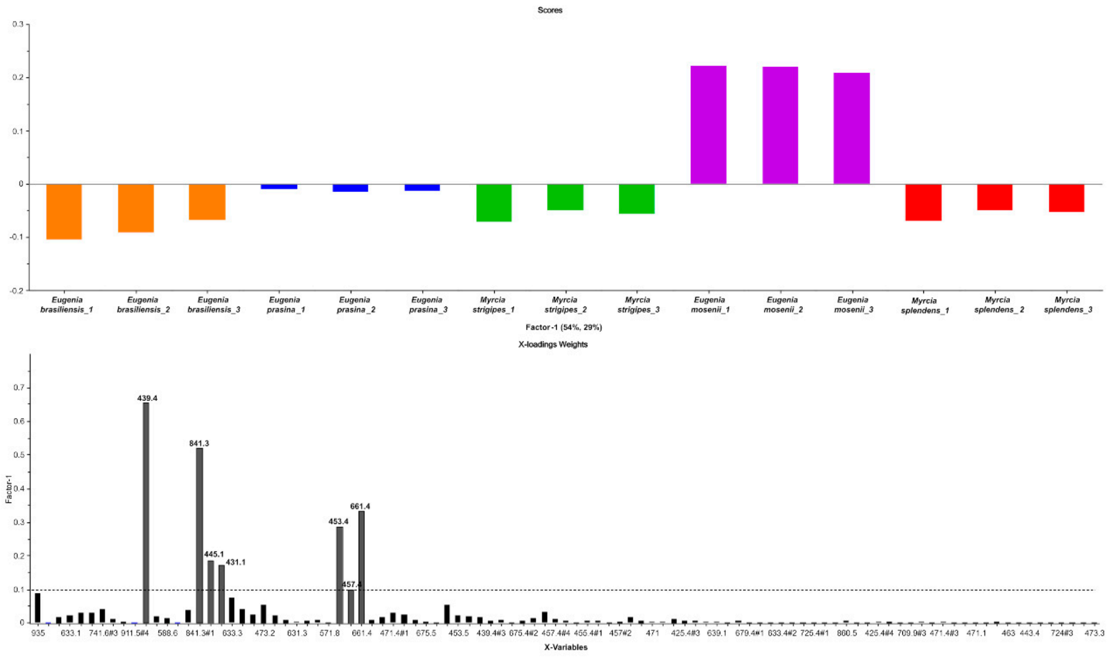

Figure S3. Scores (A), X-loadings weight (B) for the PLS model based on the LC-MS data obtained in the electrospray ionization in negative mode of the 15 samples of five species of Myrtaceae family, and the inhibition values of the extracts on the activity of the rPLpro enzyme of SARS-CoV-2. The dashed line indicates the cutoff for contributions exceeding 0.1 (loadings weights).

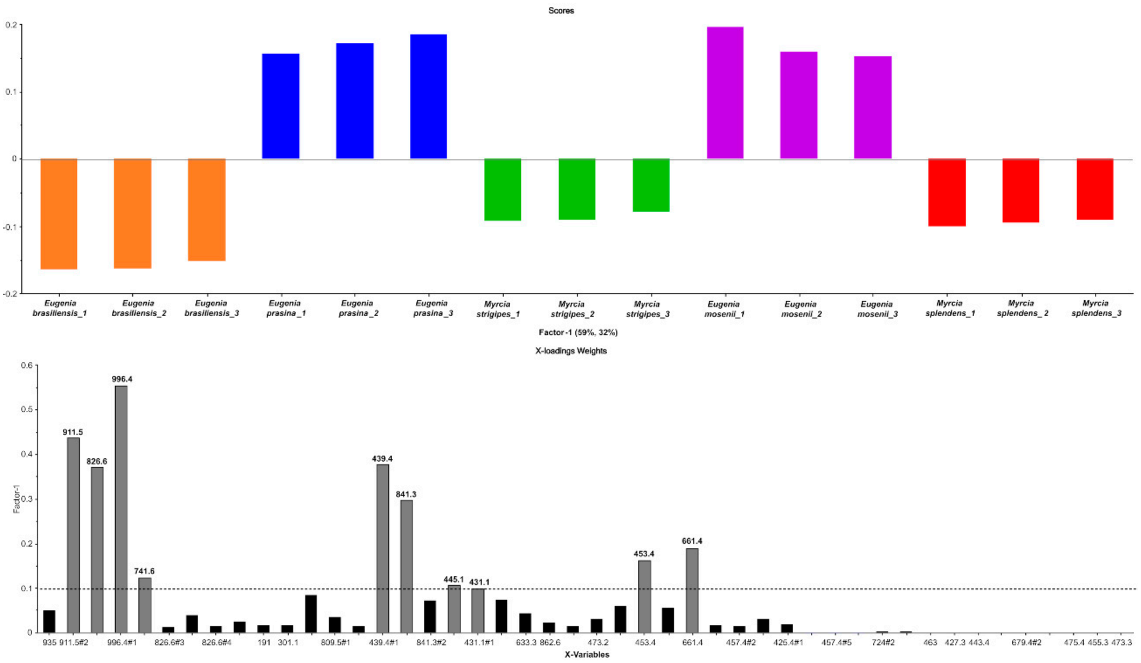

Figure S4. Feature Based Molecular Network (FBMN) of Myrtaceae extracts

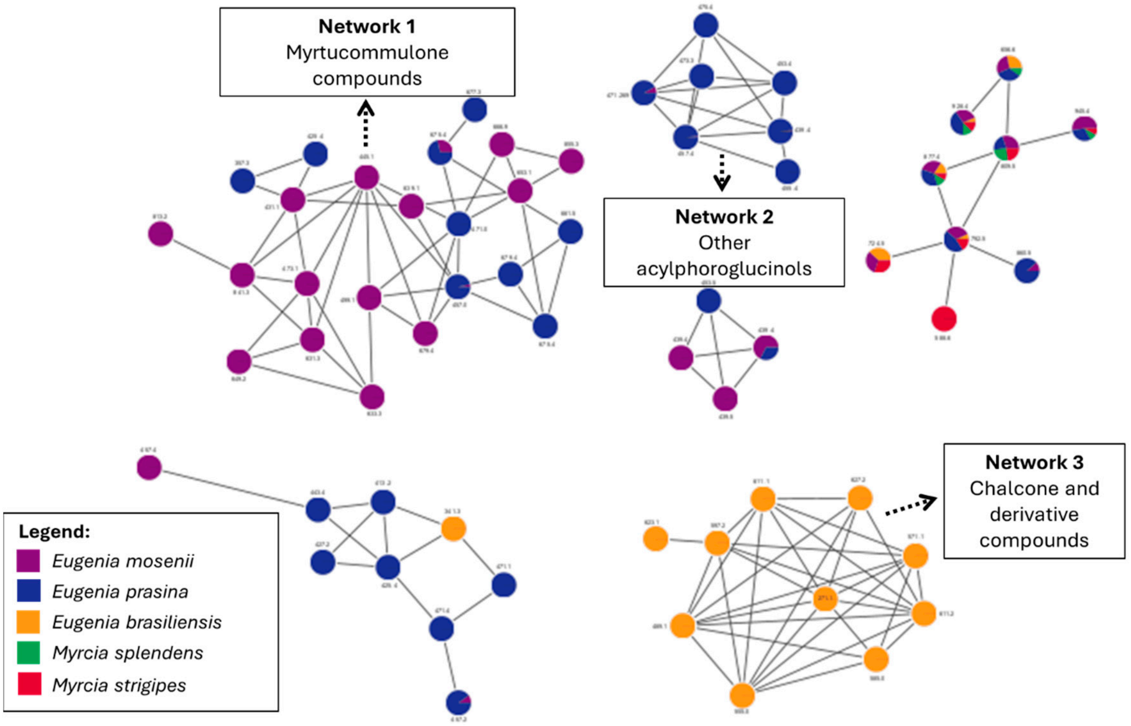

Figure S5. MS/MS spectrum of compound **1** at *m/z* 445.1 [M-H]$^{-}$ (semimyrtucommulone)

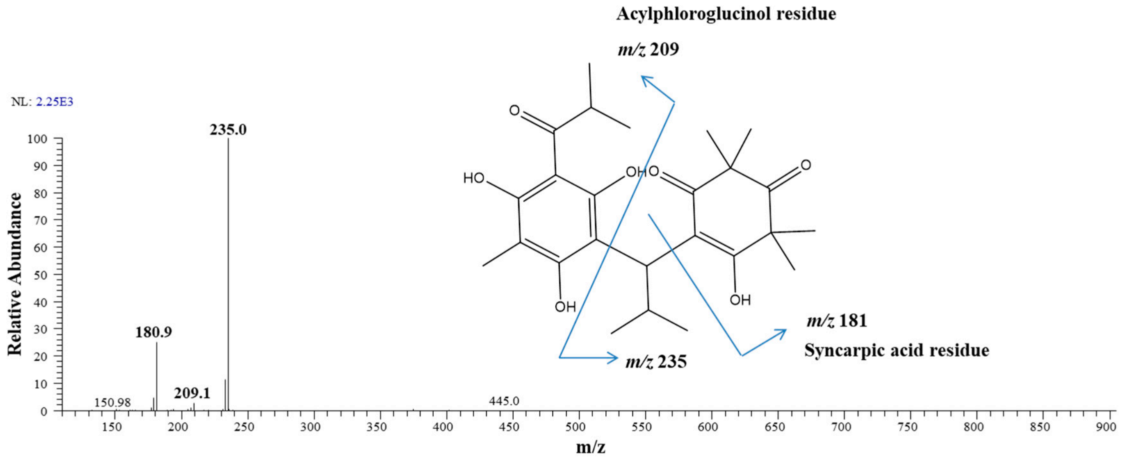

Figure S6. MS/MS spectrum of compound **2** at *m/z* 431.1 [M-H]$^{-}$ (*nor*-semimyrtucommulone)

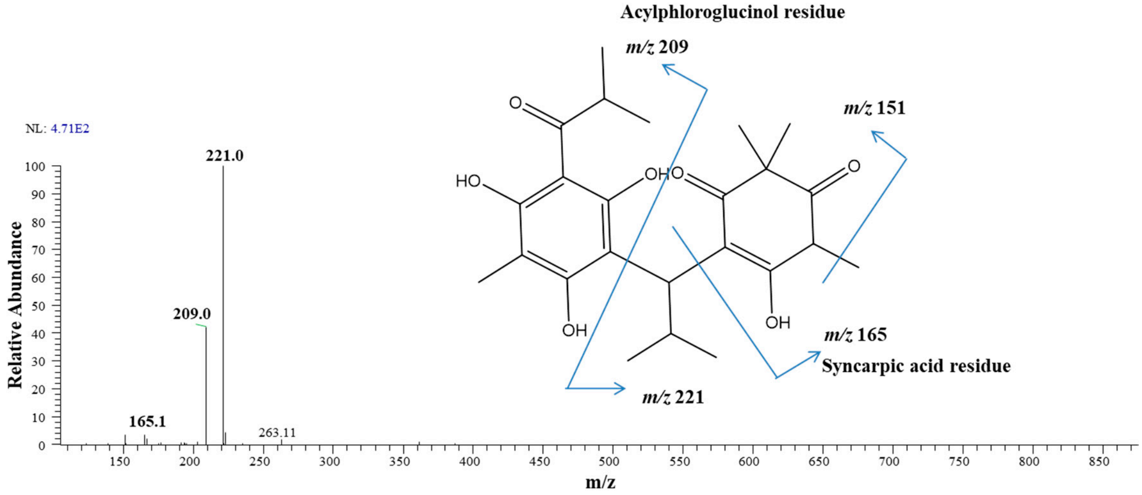

Figure S7. MS/MS spectrum of proposed compound **3** at *m/z* 473.2 [M-H]⁻

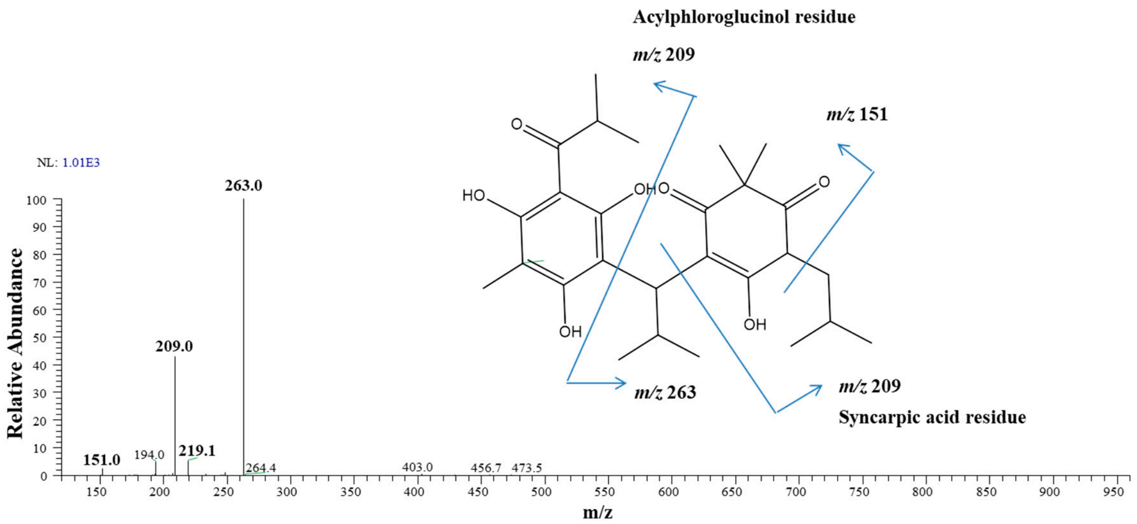

Figure S8. MS/MS spectrum of proposed compound **4** at *m/z* 633.3 [M-H]⁻

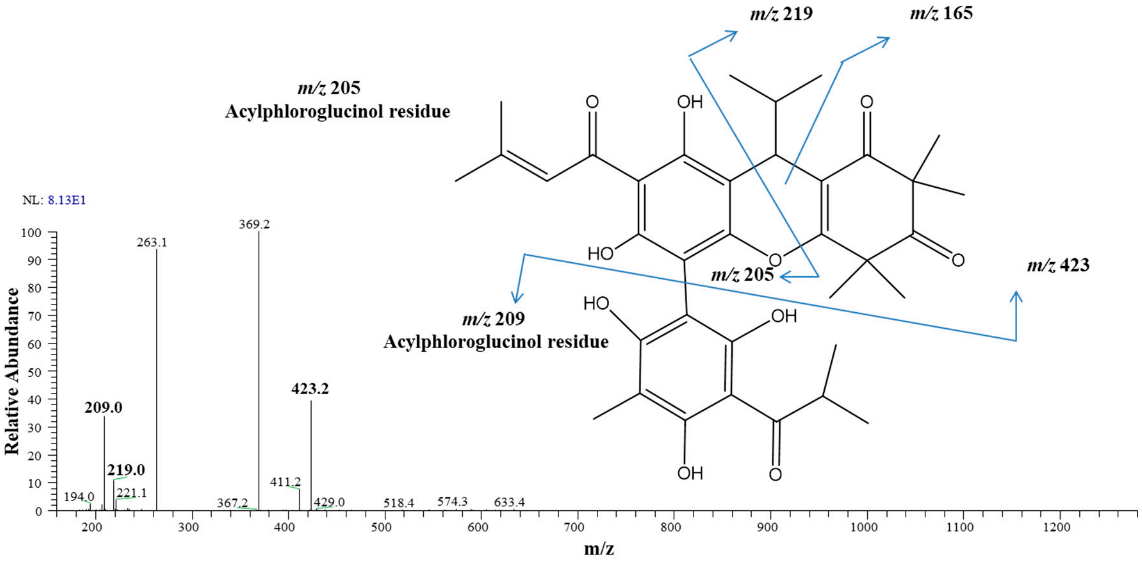

Figure S9. MS/MS spectrum of proposed compound **5** at *m/z* 841.3 $[M-H]^-$

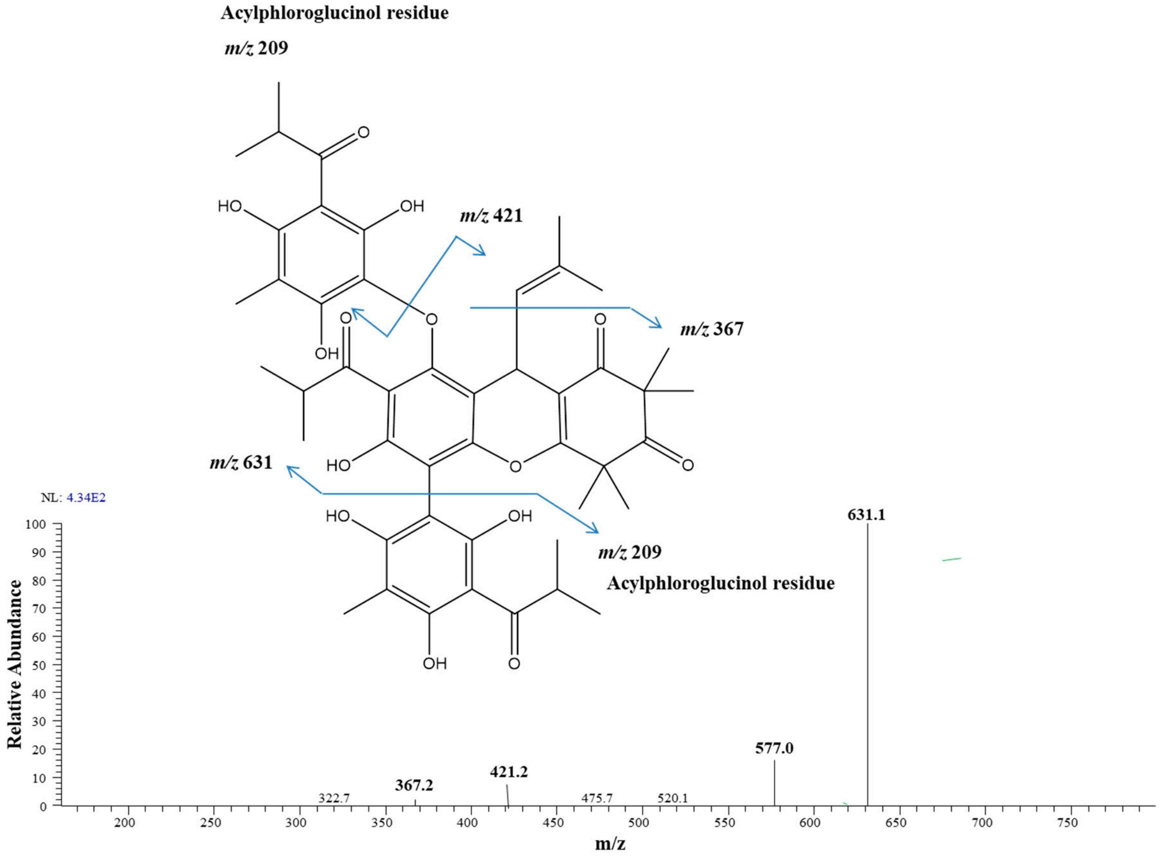

Figure S10. Molecular network of chalcone and chalcone-derivative compounds and MS/MS spectrum of *m/z* 271.1 [M-H]$^-$ (2',6'-dihydroxy-4'-methoxydihydrochalcone)

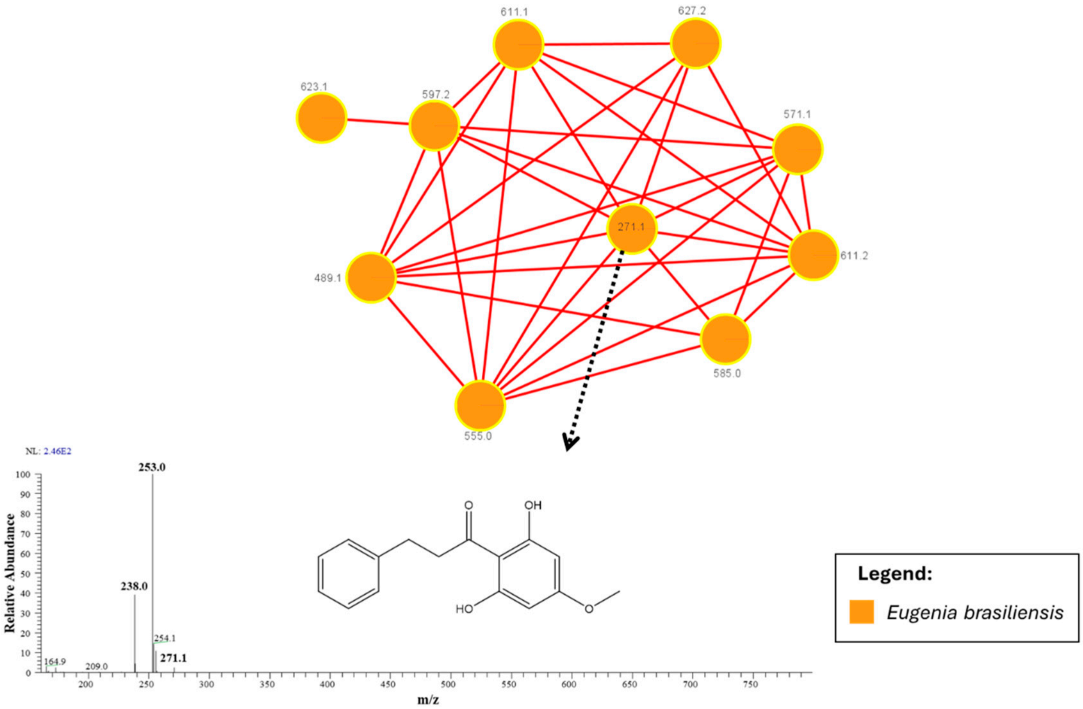

Figure S11. 2D structure of compound **5** (*m/z* 841 [M-H]$^-$) with atomic positions.

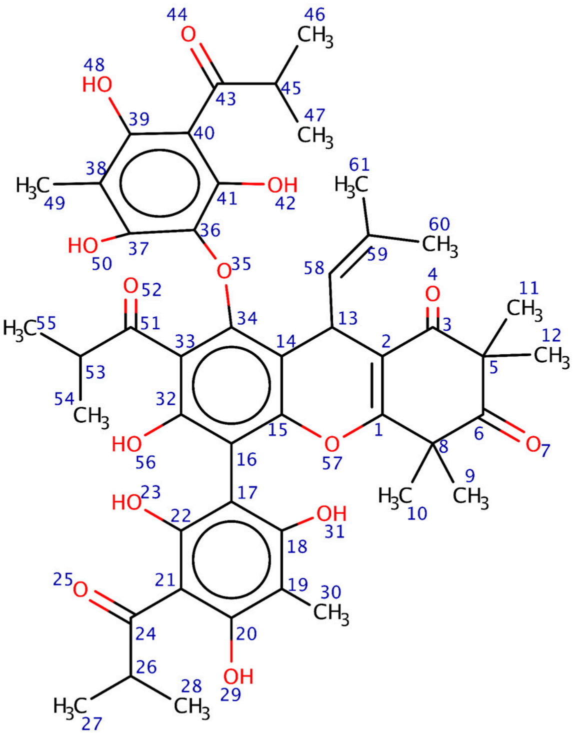

Movie S1 - Molecular dynamics results of the complex PLpro with compound 5. The protein structure is shown in gray, the BL2loop is shown in yellow and the compound 5 is shown in blue. The file is available in 10.5281/zenodo.10257486.

Movie S2 - Molecular dynamics results of the complex $3CL^{pro}$ active site with compound **5**. The protein structure is shown in gray and the compound **5** is shown in orange. The file is available in https://doi.org/10.5281/zenodo.10257448.

Movie S3 - Molecular dynamics results of the complex $3CL^{pro}$ allosteric groove with compound **5**. The protein structure is shown in gray and the compound **5** is shown in magenta. The file is available in https://doi.org/10.5281/zenodo.10257460.

Table S1 - Description of the pharmacokinetic properties of the compounds **3**, **4**, **5**, *nor*-semimyrtucommulone (**2**), and semimyrtucommulone (**1**).

	COMPOUND 3 (*m/z* 473 [M-H]-)	compound 4 (*m/z* 633 $[M-H]^-$)	compound 5 (*m/z* 841 $[M-H]^-$)	*nor*-semimyrtucommulone (2)	semimyrtucommulone (1)
**CYP1A2-INH**	Yes	Yes	Yes	Yes	Yes
**CYP1A2-SUB**	No	No	No	No	No
**CYP2C19-INH**	Yes	Yes	Yes	Yes	Yes
**CYP2C19-SUB**	No	Yes	Yes	No	No
**CYP2C9-INH**	Yes	No	Yes	Yes	Yes
**CYP2C9-SUB**	No	No	No	No	No
**CYP2D6-INH**	Yes	Yes	Yes	Yes	Yes
**CYP2D6-SUB**	Yes	Yes	Yes	Yes	Yes
**CYP3A4-INH**	Yes	Yes	Yes	Yes	Yes
**CYP3A4-SUB**	No	No	No	No	No
**CACO-2**	Yes	No	No	Yes	Yes
**BBB**	Yes	Yes	Yes	Yes	Yes
**PPB**	High	High	High	High	High
**FU (%)**	High	High	High	High	Less

CYP: Cytochrome P; Caco-2: *Cancer coli* –2; BBB: Blood–Brain Barrier; PPB: Plasma protein binding; Fu: Fraction unbound.
